# Protein kinase A modulation of Ca_V_1.4 calcium channels

**DOI:** 10.1038/ncomms12239

**Published:** 2016-07-26

**Authors:** Lingjie Sang, Ivy E. Dick, David T. Yue

**Affiliations:** 1 Calcium Signals Laboratory, Department of Biomedical Engineering, The Johns Hopkins University School of Medicine, Ross Building, Room 713, 720 Rutland Avenue, Baltimore, Maryland 21205, USA; 2 Department of Neuroscience, The Johns Hopkins University School of Medicine, Baltimore, Maryland 21205, USA

## Abstract

The regulation of L-type Ca^2+^ channels by protein kinase A (PKA) represents a crucial element within cardiac, skeletal muscle and neurological systems. Although much work has been done to understand this regulation in cardiac Ca_V_1.2 Ca^2+^ channels, relatively little is known about the closely related Ca_V_1.4 L-type Ca^2+^ channels, which feature prominently in the visual system. Here we find that Ca_V_1.4 channels are indeed modulated by PKA phosphorylation within the inhibitor of Ca^2+^-dependent inactivation (ICDI) motif. Phosphorylation of this region promotes the occupancy of calmodulin on the channel, thus increasing channel open probability (*P*_O_) and Ca^2+^-dependent inactivation. Although this interaction seems specific to Ca_V_1.4 channels, introduction of ICDI_1.4_ to Ca_V_1.3 or Ca_V_1.2 channels endows these channels with a form of PKA modulation, previously unobserved in heterologous systems. Thus, this mechanism may not only play an important role in the visual system but may be generalizable across the L-type channel family.

## Abstract (web summary)

Phosphorylation of L-type calcium Ca_V_ channels by protein kinase A is essential for several physiological events. Here, the authors show how this kinase regulates Ca_V_1.4 activity, suggesting a general regulatory mechanism for all L-type calcium channels.

## Graphical abstract

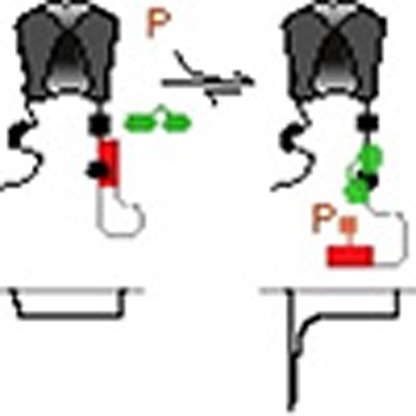

Phosphorylation, as one of the most ubiquitous forms of posttranslational modification, plays essential roles in biological functions^1^. Phosphorylation of L-type voltage-gated calcium channels by protein kinase A (PKA) is essential for normal cardiac function^2^, muscle contraction^3^ and neurological activity^4^. PKA-mediated phosphorylation underlies changes in the open probability of skeletal muscle Ca_V_1.1 and cardiac Ca_V_1.2 L-type calcium channels during the fight-or-flight response^4^,^5^, whereas phosphorylation of Ca_V_1.3 channels has been implicated in shaping neuronal action potentials^6^. However, far less is known about the effects of phosphorylation on the Ca_V_1.4 L-type channel, the main L-type channel in the retina^7^,^8^. PKA activity in the retina is robustly regulated by dopamine^9^ and dopamine fluctuates according to the circadian rhythm^10^. Thus, PKA regulation of Ca_V_1.4 could modulate visual sensitivity during the day–night cycle, making phosphorylation of Ca_V_1.4 channels a potentially important regulatory process required for normal visual function.

Similar to other L-type channels, Ca_V_1.4 channels employ a form of feedback regulation called calmodulation^11^, to precisely tune Ca^2+^ entry through these channels. Calmodulin (CaM) is known to associate with the IQ domain of the channel carboxy terminus while in the Ca^2+^-free (apo) state^12^,^13^. The presence of apoCaM on the channel IQ significantly increases channel open probability (*P*_O_) as compared with the channel in the absence of apoCaM^14^. In addition, the presence of apoCaM enables Ca^2+^-dependent inactivation (CDI), whereby Ca^2+^ binding to the resident CaM results in a decrease in channel *P*_O_^12^. Several L-type channel splice variants exploit this calmodulation effect in order to tune the biophysical properties of channels expressed in different cell types^15^,^16^,^17^. In particular, long splice variants of Ca_V_1.4 channels contain a CDI-inhibiting module (inhibitor of CDI, ICDI) within their distal C terminus^18^ (DCT), which competes with apoCaM for binding at the IQ domain^19^,^20^. By dislodging CaM, ICDI profoundly suppresses the channel *P*_O_ and eliminates CDI. We hypothesize that a phosphorylation site in ICDI might be able to modulate this interaction, thus tuning channel *P*_O_ and CDI. Moreover, ICDI is not limited to Ca_V_1.4. Other L-type calcium channels, including Ca_V_1.2 and Ca_V_1.3, have similar motifs within their DCTs, providing a broader impact for such a mechanism.

Here we set out to determine whether Ca_V_1.4 channels might indeed be modulated by PKA and what the underlying mechanism leading to such modulation might be. Remarkably, Ca_V_1.4 channels exhibit robust PKA modulation of both the current amplitude and CDI. This co-regulation of channel opening and CDI stems from a mechanism whereby the ICDI of Ca_V_1.4 channels competes with CaM binding to the channel IQ domain. Although this mechanism appears to be specific to Ca_V_1.4 channels, this form of PKA modulation can be endowed onto other L-type channels by delivering ICDI_1.4_, demonstrating a modular and partially conserved mechanism for PKA regulation across L-type channels. Moreover, we identify S1883 within ICDI_1.4_ as the phosphorylation site for this form of PKA regulation. In all, PKA modulation of Ca_V_1.4 Ca^2+^ channels, might be an important regulatory mechanism, enabling precise tuning of Ca^2+^ influx under various physiological conditions.

## Results

### PKA activation regulates full-length Ca_V_1.4 channels

**Figure 1:**
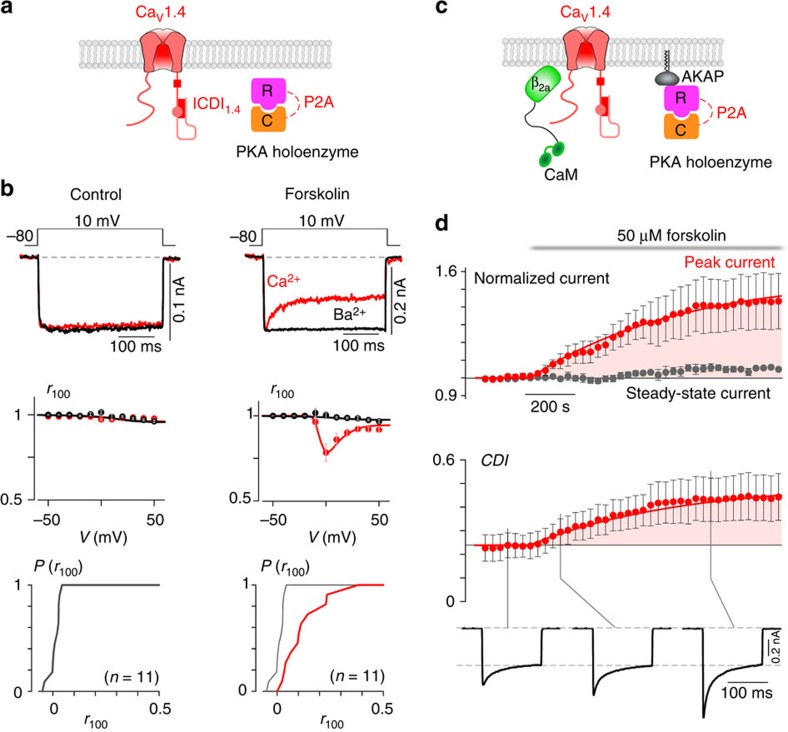
PKA regulation of full-length Ca_V_1.4 channels. (**a**) Schematic of HEK293 cells co-expressing Ca_V_1.4 channels and PKA holoenzyme (catalytic subunit, C and regulatory subunit, R, linked by a P2A sequence at the DNA level, see Methods). (**b**) Left: minimal CDI at basal PKA levels. (Top) Exemplar of whole-cell current illustrates a lack of CDI, as seen by the minimal difference in decay of Ca^2+^ (red) versus Ba^2+^ (black) current. Vertical scale bar pertains to Ca^2+^ current; Ba^2+^ current scaled downwards ∼3 × to facilitate comparison of decay kinetics, here and throughout. Middle: population data confirms minimal CDI. The fraction of peak current remaining after 100-ms (*r*_100_) is plotted versus step voltage (*V*), for mean Ba^2+^ (black) and Ca^2+^ (red) currents±s.e.m. Bottom: the tight cumulative distribution of *r*_100_ across all cells also indicates minimal CDI. Right: CDI emerges after incubation in 50 μM forskolin at 30 °C for 60 min. Format as left. Bottom right: red trace indicates a right shift and increased spread in the cumulative distribution as compared with without forskolin (grey). (**c**) Schematic of co-expression of Ca_V_1.4 channels with PKA holoenzyme, AKAP79 and β_2a_ tethered CaM, which allowed longer whole-cell patching without loss of PKA or CaM. (**d**) Time course of normalized peak current (red) and steady-state current (grey) in response to the addition of 50 μM forskolin, evoked every 30 s by depolarizations to 20 mV (top). The corresponding increase in CDI (middle) from whole-cell Ca^2+^ currents. Currents were normalized to baseline. CDI was measured as 1−*r*_100_. All error bars indicate ±s.e.m. (*n*=4 cells). Corresponding current waveforms are displayed below.

Although Ca_V_1.4 channels are known to exist in retinal cells^7^,^8^ and several other neuronal cell types^21^, they are rarely studied in their native environment. This is due in part to the difficulty in isolating and patch clamping these unique primary cells, as well as the low native open probability of the Ca_V_1.4 channels^14^. These two features make whole-cell recordings of these channels in primary cells exceedingly challenging, limiting experiments to heterologous expression systems such as HEK293 cells. Although Ca_V_1.4 channels do express in these model cells, reconstitution of PKA regulation in these cells has been challenging for other L-type calcium channels^22^,^23^,^24^. Even the strong PKA enhancement of cardiac Ca_V_1.2 L-type Ca^2+^ channels, which is readily apparent in isolated myocytes, cannot be fully reconstituted in heterologous systems^22^,^23^,^24^,^25^. We therefore adjusted our recording conditions to maximize the potential for PKA modulation within HEK293 cells (see Methods). Specifically, we co-expressed full-length, wild-type Ca_V_1.4 along with additional PKA holoenzyme (Fig. 1a). In addition, to minimize the perturbation of the cells, they were incubated with 50 μM forskolin at 30 °C for 60 min before whole-cell patch clamp recording. Indeed, these channels exhibited no CDI at baseline; however, following the forskolin incubation significant CDI was elicited, as demonstrated by the stronger decay in the Ca^2+^ (red) current as compared with the Ba^2+^ (black) current (Fig. 1b, top). Population data plotting the ratio of current remaining after 100 ms (*r*_100_) demonstrated a hallmark U-shaped dependence on voltage, further corroborating induction of CDI in Ca_V_1.4 channels in response to PKA activity (Fig. 1b, middle). Although the extent of CDI varied somewhat from cell to cell, a significant right shift in the cumulative distribution of *r*_100_ demonstrates a clear effect of forskolin on CDI (Fig. 1b, bottom, red).

Although the effect on CDI is compelling, stronger evidence would require dynamic modulation of PKA activity within a cell. Cognizant of the challenges introduced by dialysis of the cytosol during whole-cell patch clamping, we adjusted conditions to reduce this confounding factor. CaM was tethered to the β_2a_ subunit of the channel and AKAP79 was co-expressed to anchor PKA to the membrane and reconstitute the native PKA system described in primary cells^4^,^5^,^26^ (Fig. 1c). Moreover, experiments were carried out at a temperature of 30 °C, to enhance enzyme activity as compared with room temperature (see Methods). With the system thus optimized, we were indeed able to demonstrate a large dynamic PKA response. A 20 mV depolarizing step was applied for 150 ms every 30 s, allowing resolution of the current amplitude and CDI over the time course of forskolin wash-on (Fig. 1d). The addition of forskolin caused a steady increase in peak current amplitude (red), reaching a level significantly larger than the pre-forskolin current. The steady-state current, as measured after 100 ms of depolarization, did not change significantly (grey). As a result, a corresponding increase in CDI was also seen (bottom). Thus, wild-type Ca_V_1.4 channels exhibit robust PKA modulation of both channel opening and CDI.

### PKA regulation of the IQ and ICDI interaction in Ca_V_1.4

**Figure 2:**
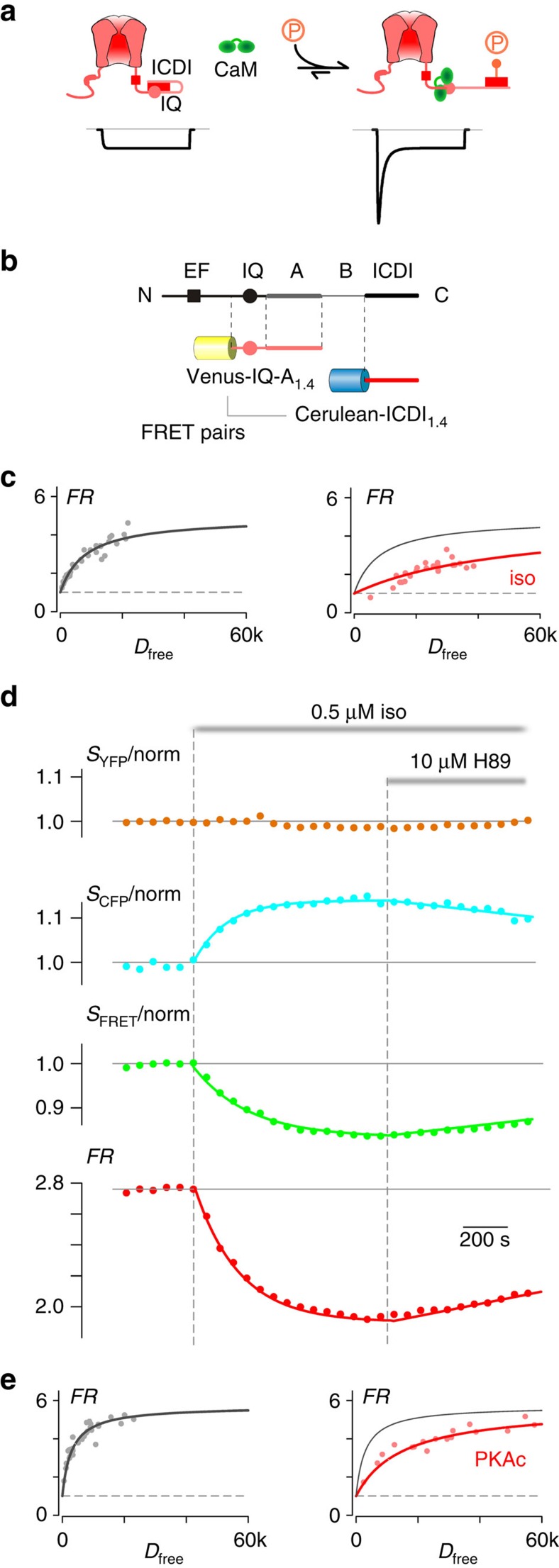
PKA regulation of the interaction between IQ and ICDI domains in Ca_V_1.4. (**a**) PKA regulation of L-type Ca^2+^ channel calmodulation. Left: ICDI module (red rectangle) on the DCT of the channel interacts with the IQ domain (pink circle) and dislodges CaM (green dumbbell). Without CaM, channels have low *P*_O_ and minimal CDI (idealized current underneath the cartoon). Right: PKA phosphorylation (orange lollipop) could weaken the IQ/ICDI interaction and allow CaM to rebind, thus increasing channel *P*_O_ and CDI. (**b**) Diagram of the C-tail of the channel illustrating the relevant FRET pairs, Venus-tagged IQ-A domains and Cerulean-tagged ICDI domain from Ca_V_1.4. (**c**) FRET binding curve for Venus-IQ-A_1.4_ paired with Cerulean-ICDI_1.4_ in aGPVMs (grey). Isoproterenol (0.5 μM iso; red) decreases the relative binding affinity. Each point indicates a single cell. The control binding curve is replicated as the grey curve in the right-hand panel here and throughout. FR, FRET ratio; *D*_free_, relative concentration of unbound Cerulean-tagged ICDI_1.4_. (**d**) Kinetics of normalized fluorescent signals from YFP channel (*S*_YFP_), CFP channel (*S*_CFP_), FRET channel (*S*_FRET_) and calculated FR from an exemplar aGPVM expressing Venus-IQ-A_1.4_ and Cerulean-ICDI_1.4_, with 0.5 μM isoproterenol and 10 μM H89 added as indicated. Fluorescent signals were normalized to baseline. (**e**) FRET binding curve for Venus-IQ-A_1.4_ paired with Cerulean-ICDI_1.4_ in HEK293 cells (grey). Overexpression of PKAc (red) reduces the binding.

The effects of PKA activity on channel *P*_O_ and CDI is reminiscent of a previous study whereby CaM was enriched near the Ca_V_1.3_S/1.4DCT_ chimeric L-type channel via rapamycin-induced dimerization^14^. Thus, a possible underlying mechanism of PKA modulation of Ca_V_1.4 channels may involve a similar CaM centric mechanism. In particular, the IQ region of calcium channels is known to bind to apoCaM, enhancing channel *P*_O_ and enabling CDI upon Ca^2+^ entry. In addition, the ICDI domain in certain L-type Ca^2+^ channels is known to bind to the IQ region of the channel, displacing CaM and reducing the CDI and *P*_O_ of the channel^14^,^20^. We hypothesized that the PKA modulation of Ca_V_1.4 channels might use this regulatory mechanism, such that ICDI bound to the IQ region at baseline might be displaced by CaM following PKA activation (Fig. 2a). With CaM replacing ICDI on the IQ region of the channel, the channel *P*_O_ would be increased and CDI enabled^14^. We thus sought to assess whether the interaction between the ICDI and IQ domains might be modulated by PKA phosphorylation. To this end, we used a live-cell fluorescence resonance energy transfer (FRET) two-hybrid assay, pairing Venus-tagged IQ domains against Cerulean-tagged ICDI domains from Ca_V_1.4 channels. Although not required for CaM binding^27^, the downstream A-region^18^,^20^ was included within our IQ construct (IQ-A), as it contributes to the binding between the IQ and ICDI domains^28^. Cognizant that full-bore PKA signalling is best conserved within certain native rather than model cells^2^,^22^,^23^,^25^,^29^, we performed FRET assays in adult guinea pig ventricular myocytes (aGPVMs), which are renowned for their strong PKA signalling^2^. The FRET pairs were transduced into aGPVMs via adenoviral vectors, allowing rapid and tunable expression within acute (1 day) myocytes^30^.

Indeed, FRET two-hybrid measurements in aGPVMs yielded a robust binding curve (Fig. 2c, black and Supplementary Table 1), indicating a clear interaction between these two channel domains. Remarkably, however, this interaction is largely disrupted when PKA is activated by the addition of 0.5 μM isoproterenol (Fig. 2c, red). Moreover, this PKA effect can be elicited as a dynamic response within an exemplar myocyte (Fig. 2d). The application of 0.5 μM isoproterenol decreases the FRET ratio (FR) exponentially (red), indicating a disruption in binding. Of note, the individual CFP and FRET signals have corresponding changes, whereas the YFP signal remains relatively stable when independently excited, indicating a genuine FRET response rather than an artefact due to isoproterenol acting directly on the fluorophores. Importantly, the decrease in FR can be reversed by inhibiting PKA via addition of the PKA inhibitor H89 (Fig. 2d), demonstrating a PKA-specific effect.

The discovery that the binding between IQ and ICDI domains in Ca_V_1.4 can be attenuated by PKA activation in aGPVMs is compelling; however, the functional implications of this modulation could best be probed in a heterologous expression system where full control of channel and interacting partners is possible^13^. We therefore sought to reconstruct the PKA regulation of IQ binding with ICDI in HEK293 cells. We first attempted to replicate the PKA modulation of this interaction through the application of forskolin; however, the effect of forskolin was minimal (Supplementary Fig. 1a). Reasoning that HEK293 cells may have decreased endogenous PKA signalling as compared with aGPVMs, we overexpressed the constitutively active catalytic subunit of PKA (PKAc) and found that the binding of Ca_V_1.4 IQ-A with ICDI was indeed reduced in HEK293 cells (Fig. 2e), thus rationalizing the overexpression of PKA holoenzyme in our whole-cell experiments (Fig. 1). Overall, the binding of IQ-A and ICDI can be dynamically modulated by PKA phosphorylation in a reversible manner, supporting the hypothesis that modulation of this interaction underlies the PKA regulation of Ca_V_1.4 channels.

### Lack of ICDI-mediated PKA regulation of Ca_V_1.3 and Ca_V_1.2

**Figure 3:**
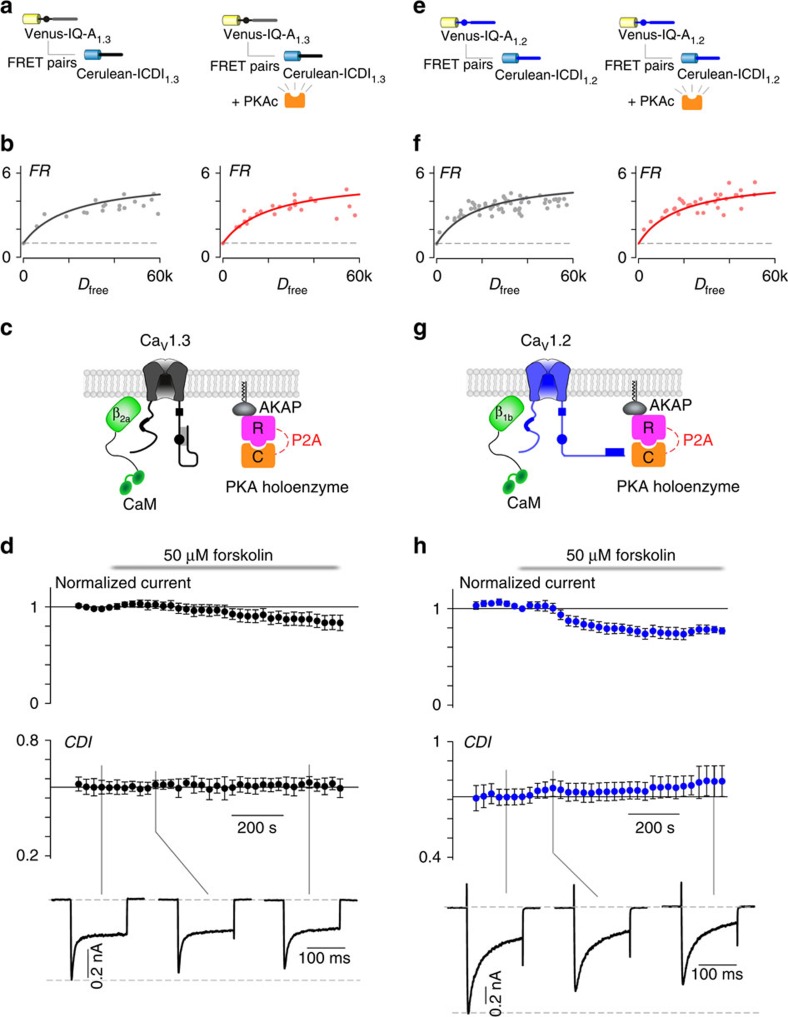
PKA does not regulate the interaction of IQ and ICDI domains in Ca_V_1.3 or Ca_V_1.2 channels. (**a**,**b**) Overexpression of PKAc does not change the binding between IQ-A_1.3_ and ICDI_1.3_ in HEK293 cells. (**c**) Schematic of co-expression of Ca_V_1.3 with PKA holoenzyme, AKAP79 and β_2a_ tethered CaM, which allowed longer whole-cell patching without loss of PKA or CaM. (**d**) Time course of normalized peak current (top) and CDI (middle) in response to the addition of 50 μM forskolin (*n*=5 cells). Corresponding current waveforms are displayed below. (**e**, **f**) Overexpression of PKAc does not change binding between IQ-A_1.2_ and ICDI_1.2_ in HEK293 cells. (**g**) Schematic of co-expression of Ca_V_1.2 with PKA holoenzyme, AKAP79 and β_1b_ tethered CaM, which allowed longer whole-cell patching without loss of PKA or CaM. (**h**) Time course of normalized peak current (top) and CDI (middle) in response to the addition of 50 μM forskolin (*n*=4 cells). Corresponding current waveforms are displayed below.

The ICDI domain of Ca_V_1.4 exhibits significant sequence homology to the ICDI domain of other L-type Ca^2+^ channels. In fact, the ability of ICDI to compete with CaM for binding to the IQ domain of the channel has been demonstrated for both Ca_V_1.3 and Ca_V_1.2 channels^14^,^20^,^28^. We therefore wondered whether these channels may also employ a similar mechanism of PKA phosphorylation. To test this, we used the FRET two-hybrid binding assay to probe for interactions between the IQ and ICDI regions of Ca_V_1.3 and 1.2 channels (Supplementary Table 1). As expected from previous studies, the ICDI_1.3_ bound well to IQ-A_1.3_; however, PKA phosphorylation failed to modulate this interaction in either HEK293 cells (Fig. 3b) or aGPVMs (Supplementary Fig. 2). Moreover, even with the enhanced PKA expression system used for Ca_V_1.4 channels (Fig. 1), forskolin failed to induce a change in CDI or current amplitude of Ca_V_1.3 channels in HEK293 cells (Fig. 3c,d). Nearly identical results were seen for Ca_V_1.2 channels, where significant binding between the ICDI and IQ domains is unperturbed by PKA activation (Fig. 3e,f and Supplementary Fig. 2) and no modulation of CDI or current amplitude can be seen in the heterologous expression system (Fig. 3g,h). Thus, despite the sequence similarity of IQ and ICDI domains across Ca_V_1.2, 1.3 and 1.4, the PKA regulatory mechanism is unique to Ca_V_1.4.

### Engineering ICDI-mediated PKA regulation in Ca_V_1.3 channels

**Figure 4:**
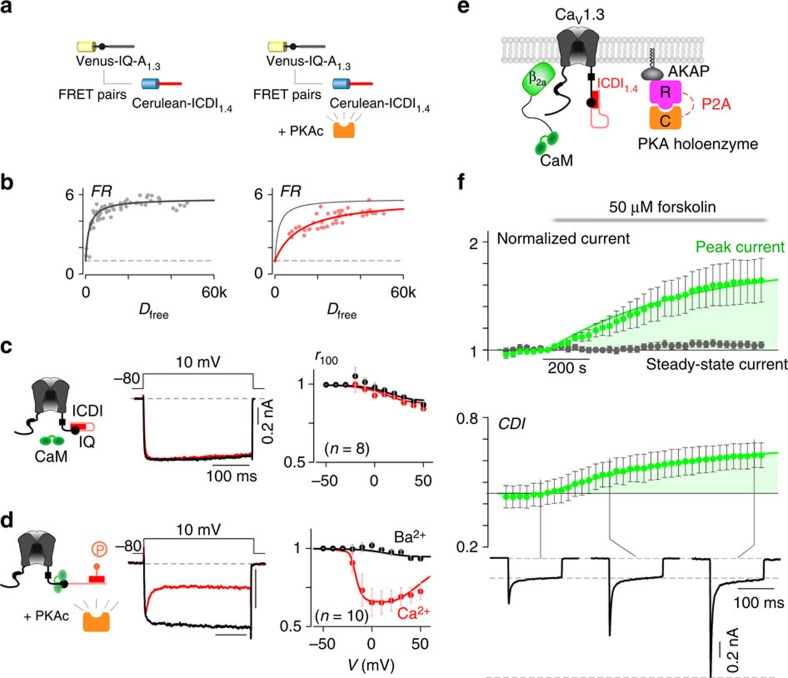
PKA regulation of chimeric Ca_V_1.3_S/1.4DCT_ channels in HEK293 cells. (**a**) FRET binding assays between Venus-IQ-A_1.3_ and Cerulean-ICDI_1.4_ demonstrating the effect of PKAc overexpression. (**b**) PKAc reduces the binding between IQ-A_1.3_ and ICDI_1.4_. (**c**) Cartoon (left) of the chimeric channel Ca_V_1.3_S/1.4DCT_ made by attaching the DCT of Ca_V_1.4 (including ICDI, red) to a truncated Ca_V_1.3. Exemplar whole-cell current (middle) and population data (right) illustrates a lack of CDI. (**d**) Overexpression of PKAc (left) restored CDI of this chimeric channel, showing a strong decay of Ca^2+^ current (red) compared with Ba^2+^ current (black). The classic U-shaped voltage dependence of CDI is seen in the population data (right). (**e**) Schematic of co-expression of Ca_V_1.3_S/1.4DCT_ with PKA holoenzyme, AKAP79 and β_2a_ tethered CaM, which allowed longer whole-cell patching without loss of PKA or CaM. (**f**) Time course of normalized peak current (green) and steady-state current (grey) in response to the addition of 50 μM forskolin, evoked every 30 s by depolarizations to 20 mV (top). The corresponding increase in CDI (middle) from whole-cell Ca^2+^ currents. Currents were normalized to baseline. CDI was measured as 1−*r*_100_. All error bars indicate ±s.e.m. (*n*=6 cells). Corresponding current waveforms are displayed below.

To test our hypothesis that PKA regulation of Ca_V_1.4 channels is the direct result of a phosphorylation-induced change in binding affinity between the channel IQ region and the ICDI domain, we sought to recreate this form of regulation in a channel which does not natively display such regulation. We thus sought to endow Ca_V_1.3 channels with ICDI-mediated PKA regulation by manipulating the ICDI domain. We began by pairing the Ca_V_1.3 channel with the ICDI domain from Ca_V_1.4 (ICDI_1.4_) as previous studies have demonstrated an alteration in both the CDI and channel *P*_O_ as a result of this interaction^20^,^28^. To test whether the interaction between the Ca_V_1.3 IQ domain (IQ-A_1.3_) and ICDI_1.4_ could be modulated by PKA, we again used FRET two-hybrid to characterize the interaction of the two channel segments. Indeed, FRET two-hybrid measurements in aGPVMs yielded a robust binding curve (Supplementary Fig. 3 and Supplementary Table 1), indicating a clear interaction between these two channel domains. This interaction, however, is largely disrupted when PKA is activated by the addition of 0.5 μM isoproterenol (red), an effect that can also be elicited as a dynamic response within an exemplar myocyte (Supplementary Fig. 3) and can be reversed either by washing off isoproterenol or by inhibiting PKA via addition of either the wide-spectrum kinase inhibitor staurosporine or the PKA inhibitor H89 (Supplementary Fig. 3). Moreover, this PKA effect can be recreated in HEK293 cells (Fig. 4b), indicating a robust effect. Thus, the binding of IQ-A_1.3_ and ICDI_1.4_ can be dynamically modulated by PKA phosphorylation.

Given the ability of ICDI_1.4_ to compete with CaM for the Ca_V_1.3 channel IQ region^20^, we tested whether disruption of IQ/ICDI binding by PKA might affect channel CDI. We used the chimeric Ca_V_1.3_S/1.4DCT_ channel^14^,^20^ (Ca_V_1.3 with the DCT replaced by that of Ca_V_1.4) as introduction of ICDI_1.4_ in this channel is known to ablate the CDI of wild-type Ca_V_1.3 (ref. 20), as seen by the minimal difference in the decay of Ca^2+^ (red) versus Ba^2+^ (black) current (Fig. 4c). However, if PKAc is now overexpressed in these cells, CDI is restored (Fig. 4d). Population data again demonstrates a hallmark U-shaped dependence on voltage, further corroborating a restoration of CDI in the chimeric channel due to PKA activity. To confirm that this effect can be induced by activating PKA, we co-expressed the chimeric Ca_V_1.3_S/1.4DCT_ channel with PKA holoenzyme and incubated the cells with 50 μM forskolin at 30 °C for 30 min before whole-cell patch clamp recording. Indeed, incubation with forskolin restored a significant amount of CDI as compared with that without forskolin (Supplementary Fig. 4). This restoration of CDI is indicative of a shift in the binding of the IQ region from ICDI_1.4_ back to CaM in the phosphorylated state (Fig. 2a).

Based on our hypothesis (Fig. 2a), phosphorylation of ICDI by PKA should increase the channel *P*_O_, in addition to restoring CDI. To observe the concurrent increase in *P*_O_ and CDI, we added 50 μM forskolin, while recording whole-cell Ca^2+^ currents at a bath temperature of 30 °C. The addition of forskolin caused a steady increase in peak current amplitude (Fig. 4f, green), reaching a level several fold larger than the pre-forskolin current. At the same time, the steady-state current remaining after 100 ms was remarkably stable (grey), resulting in an increase in CDI. This outcome is consistent with a previous study, whereby CaM was enriched near the Ca_V_1.3_S/1.4DCT_ channel via rapamycin-induced dimerization^14^, further substantiating the hypothesis that PKA activation results in increased CaM binding to the channel IQ due to a reduction in the affinity of IQ_1.3_ with ICDI_1.4_.

### ICDI_1.4_ as a modular phospho-switch for Ca_V_1.2 channels

**Figure 5:**
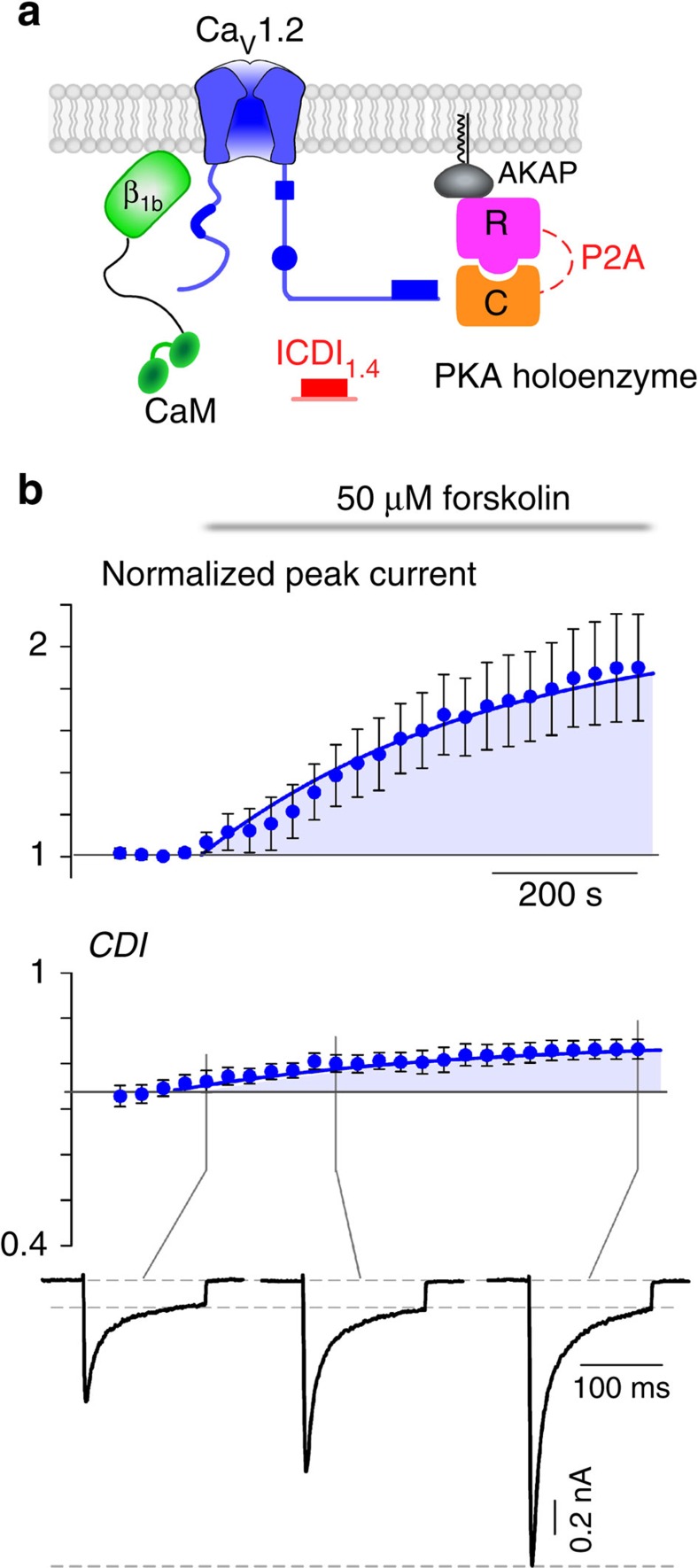
Synthetic PKA regulation of Ca_V_1.2 in HEK293 cells. (**a**) Schematic of full-length Ca_V_1.2 channel, PKA holoenzyme (R and C) and ICDI_1.4_ peptide, along with β_1b_ tethered CaM and AKAP79. (**b**) Time course of normalized peak current (top) and CDI (middle), from whole-cell Ca^2+^ currents, evoked every 30 s by depolarizations to 20 mV. Currents were normalized to baseline. CDI was measured as 1−*r*_100_. All error bars indicate ±s.e.m. (*n*=6 cells). Corresponding current waveforms are displayed below.

Beyond Ca_V_1.3, ICDI_1.4_ was also able to bind to IQ-A_1.2_ in a PKA-sensitive manner (Supplementary Fig. 2). We wondered whether this ICDI_1.4_ module could serve as a synthetic phospho-switch for Ca_V_1.2 Ca^2+^ channels, able to be plugged into a system by co-expression as a cytosolic peptide. Such synthetic modulation of the Ca_V_1.2 channel may be of particular interest, as these channels are known to undergo a large increase in current amplitude in response to PKA activation in myocytes; however, this effect is difficult to replicate in recombinant systems^22^,^23^. We therefore sought to use ICDI_1.4_ to generate PKA modulation of Ca_V_1.2 channels in HEK293 cells. To maintain the properties of cardiac Ca_V_1.2 channels as much as possible in our recombinant system, we co-expressed ICDI_1.4_ as a peptide along with the full-length cardiac Ca_V_1.2 channel and used the β_1b_ Ca^2+^ channel subunit, which more closely recapitulates the inactivation profile found in native cardiac myocytes^29^ (Fig. 5a). As before, CaM was linked to the β-subunit and AKAP was co-expressed so as to reduce dialysis of the key elements. Under these conditions, application of 50 μM forskolin largely increased the peak amplitude (Fig. 5b), similar to the native PKA upregulation of cardiac Ca_V_1.2 channels (Supplementary Fig. 5). In addition, an increase in CDI was seen even in the presence of significant voltage-dependent inactivation due to the use of the β_1b_-subunit^31^. Thus, the effect of ICDI_1.4_ on channel regulation demonstrated in the chimeric Ca_V_1.3_S/1.4DCT_ channel appears to be generalizable to other L-type Ca^2+^ channels. Moreover, ICDI_1.4_ need not be covalently attached to the channel, but rather is capable of acting as a diffusible CDI/*P*_O_ switch, which can be turned off by PKA phosphorylation.

### Identification of the PKA phosphorylation site within Ca_V_1.4

**Figure 6:**
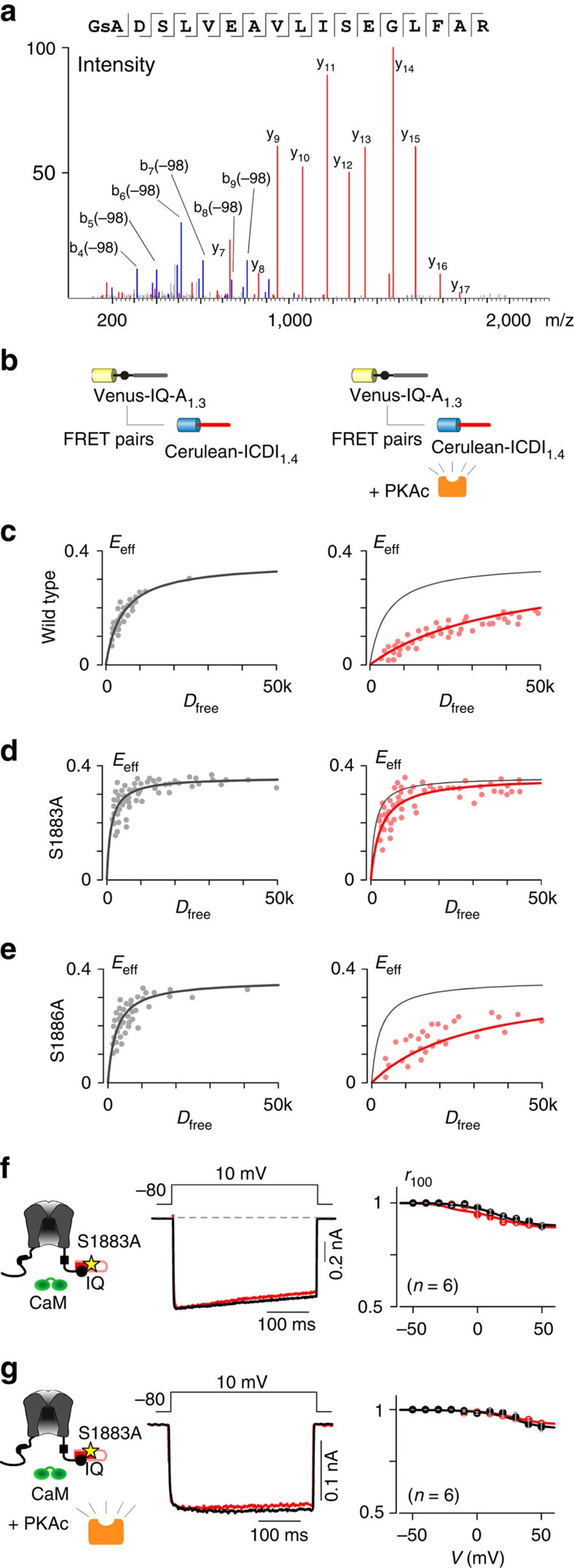
Identification of the PKA phosphorylation site within ICDI_1.4_. (**a**) The mass spectra of the ICDI_1.4_ peptide, showing phosphorylation at S1883. (**b**) Flow-cytometric FRET binding assays (**c**–**e**) between Venus-IQ-A_1.3_ and Cerulean-ICDI_1.4_ demonstrating the modulation of binding due to PKAc overexpression. (**c**) Overexpression of PKAc reduced binding between IQ-A_1.3_ and wild-type ICDI_1.4_. (**d**) Overexpression of PKAc did not affect binding between IQ-A_1.3_ and ICDI_1.4_ harboring an S1883A mutation. (**e**) Overexpression of PKAc reduced binding between IQ-A_1.3_ and ICDI_1.4_ harbouring an S1886A mutation. (**f**) Similar to wild-type Ca_V_1.3_S/1.4DCT_ channels (Fig. 4c), Ca_V_1.3_S/1.4DCT_ channels containing the S1883A mutation had minimal CDI. (**g**) Unlike wild-type Ca_V_1.3_S/1.4DCT_ (Fig. 4d), Ca_V_1.3_S/1.4DCT_ channels with the S1883A mutation did not exhibit increased CDI with overexpression of PKAc.

Thus far, we have demonstrated that elevated PKA activity correlates with increased peak current amplitude and CDI of L-type Ca^2+^ channels in the presence of ICDI_1.4_. We hypothesized that this effect is due to direct phosphorylation of ICDI_1.4_; however, an indirect effect due to phosphorylation of an unknown ICDI-interacting element could produce a similar result. As a first step towards identifying the site, we used the bioinformatics tool pkaPS^32^ to predict potential PKA phosphorylation sites within ICDI_1.4_. From this prediction algorithm, S1883 appeared to be the most probable site (Supplementary Fig. 6). As this motif is not easily recognized by currently available phospho-specific antibodies, we employed mass spectroscopy to identify phosphorylation sites within ICDI_1.4_. To induce phosphorylation, a histidine-tagged ICDI_1.4_ peptide was co-expressed with the PKA holoenzyme in HEK293 cells and subjected to 1 h of forskolin treatment. This peptide was purified using the histidine tag and analysed by mass spectroscopy, which revealed that S1883 is indeed phosphorylated (Fig. 6a and Supplementary Fig. 7); however, an additional phosphorylation site at S1886 could not be ruled out (see Methods).

Having established S1883 as a potential PKA phosphorylation site, we sought to determine whether this site is responsible for the functional modulation of the channel. To confirm that the site is required for the PKA modulation of binding between L-type channel IQ regions and ICDI_1.4_, we used a flow-cytometer-based high-throughput platform for live-cell FRET two-hybrid binding, which was recently developed by our lab^33^. As seen by traditional FRET two-hybrid binding (Fig. 4b), overexpression of PKAc reduced the binding affinity of Venus-tagged IQ-A_1.3_ and Cerulean-tagged ICDI_1.4_ peptides (Fig. 6b,c and Supplementary Table 2). However, when S1883 within ICDI_1.4_ was mutated to alanine, the PKA modulation of the interaction was abolished (Fig. 6d). In contrast, if S1886 was mutated to alanine, PKA regulation was preserved (Fig. 6e). Finally, we probed the functional effect of the S1883A mutation via whole-cell patch clamp recording. The Ca_V_1.3_S/1.4DCT_ chimeric channel was used for these experiments, as it expresses well and exhibits robust and consistent PKA modulation. Mutation of S1883 to alanine had no effect on channel properties in the absence of PKA (Fig. 6f) and overexpression of PKAc failed to alter CDI (Fig. 6g), confirming that S1883 is a novel and functional PKA phosphorylation site within the Ca_V_1.4 ICDI domain.

## Discussion

PKA phosphorylation of ICDI_1.4_ has significant, previously unreported effects on wild-type Ca_V_1.4 channels. Such PKA regulation may have important physiological consequences. Ca_V_1.4 is the main L-type Ca^2+^ channel in the retina^7^,^8^. Unfortunately, the diminutive size of the photoreceptors and small *P*_O_ of Ca_V_1.4 channels makes direct measurements of PKA modulation within this native system exceedingly challenging. Nonetheless, the creation of a robust PKA system in HEK293 cells has enabled new mechanistic insight. We now know that PKA phosphorylation of ICDI_1.4_ disrupts IQ binding, allowing CaM to bind the IQ region, thus increasing channel *P*_O_ and CDI. Such a mechanism may well be at play in the retina. Ca^2+^ currents within photoreceptors exhibit a modest amount of CDI, which had previously been attributed to a mixture of Ca_V_1.4 channel splice variants^17^,^34^. Here we have demonstrated that even the long splice variant, containing ICDI_1.4_, is capable of supporting CDI under elevated PKA activation and probably contributes to the CDI seen in these cells. Moreover, ICDI_1.4_ is highly expressed in the retina (Supplementary Fig. 8a) where PKA activity is robustly regulated by dopamine^9^. As the dopamine fluctuations in the retina occur according to the circadian rhythm, they exhibit oscillations with periods lasting for many hours^10^ rationalizing the relatively slow kinetics of Ca_V_1.4 regulation by PKA (Fig. 1d). Thus, PKA regulation of Ca_V_1.4 could modulate visual sensitivity during the day–night cycle. In fact, channelopathic mutations in Ca_V_1.4, which cause premature truncation before the ICDI domain display increased current amplitudes, augmented CDI and cause congenital stationary night blindness^19^,^35^. We therefore tested the congenital stationary night blindness channels Ca_V_1.4_K1591X_ in HEK cells and found that the channels do indeed exhibit significant CDI at baseline and have no response to 50 μM forskolin (Supplementary Fig. 8b,c) as predicted by the loss of the ICDI domain and S1883. Thus, PKA-mediated phosphorylation of the ICDI domain may play a critical role in low-light adaptation and normal visual function.

Beyond Ca_V_1.4 channels, ICDI_1.4_ was able to endow chimeric Ca_V_1.3 and 1.2 channels with robust PKA modulation, demonstrating a generalizable mechanism for regulation of L-type channel CDI and *P*_O_. Moreover, ICDI_1.4_ was also able to act as a synthetic phospho-switch when co-expressed as a separate peptide with Ca_V_1.2 channels. This synthetic PKA modulation of channel opening and CDI could be a valuable tool to study the physiological role of CDI and *P*_O_, as this small peptide could be easily transduced into native cells. Moreover, co-expression of this peptide at basal PKA levels mimics the properties of certain long Ca^2+^ channel splice variants^14^, whereas elevation of PKA activity reverts the phenotype of these channels to that of a short splice variant. As such, this tool could aid in understanding the functional effects of alterations in splice variant expression in different cell types.

Such a synthetic phospho-switch may have added value beyond that of a novel tool for regulating L-type Ca^2+^ channels. It may shed light on the mechanism underlying native PKA regulation of L-type Ca^2+^ channels. In particular, the regulation of cardiac Ca_V_1.2 channels by PKA is a critical feature of the fight-or-flight response, but the full mechanistic pathway underlying this regulation remains under debate^23^,^24^,^25^,^36^. Although PKA activation readily increases Ca_V_1.2 current by several fold in adult cardiomyocytes, such large PKA upregulation is not often observed in heterologous systems such as HEK293 cells^22^,^23^, even in the presence of exogenous PKA holoenzymes (Fig. 3g,h). It appears then that there must be a critical element in myocytes, which is lacking in recombinant systems^22^,^25^. Given the ability of ICDI_1.4_ to modulate Ca_V_1.2 channel *P*_O_ in a phosphorylation-dependent manner, we wonder whether the missing element in myocytes may employ a similar strategy. Thus, searching for proteins capable of binding channel IQ domains may prove a useful approach.

To ensure the presence of all key elements of the PKA pathway within our binding assay, we implemented quantitative live-cell FRET two-hybrid binding in acutely isolated aGPVMs. This system enabled us to probe the PKA modulation of IQ/ICDI_1.4_ interactions, a phenomenon that was not initially observable in HEK293 cells (Supplementary Fig. 1a). This failure of forskolin to modulate the IQ/ICDI_1.4_ interactions in HEK293 cells at baseline points to an important limitation of these cells. Although these cells have proven quite useful for studying many types of PKA modulation including HERG^37^, K_V_1.1 (ref. 38) and nicotinic receptors^39^, they appear less ideal for studying the modulation of L-type Ca^2+^ channels. It therefore seems likely to be that these phosphorylation targets have different thresholds for PKA modulation and model systems in which PKA is sufficient for one application may not be suitable for all target proteins.

In all, we have demonstrated that the repertoire of PKA regulation of L-type Ca^2+^ channels extends to Ca_V_1.4 channels expressed in a heterologous system. The identification of the phosphorylation site within the Ca_V_1.4 ICDI domain fits well with a mechanism in which PKA-mediated phosphorylation alters the binding affinity between the IQ and ICDI domains of Ca_V_1.4, thus tuning the extent of CDI and channel *P*_O_ via competitive displacement of CaM. Although conformation of this effect in a native system remains to be demonstrated, the distribution of Ca_V_1.4 channels in the retina points to a potentially important role in the visual system and in tuning the circadian rhythm. Overall, this modulatory scheme may represent a general mechanism by which various L-type channels could be modulated by yet-to-be-identified competitors of CaM.

## Methods

### Molecular biology

The Ca_V_1.3 channel (in pcDNA6) was derived from the rat brain variant (AF307009.1)^40^. Ca_V_1.2 (in pGW) is identical to rabbit NM001136522 (ref. 41) and Ca_V_1.4 (in pCDNA3) is the human clone corresponding to NM00718 (ref. 14). Ca_V_1.3_S/1.4DCT_ (in pcDNA6) was made by fusing with the DCT of Ca_V_1.4 to the Ca_V_1.3 channel backbone (truncated after the IQ domain)^20^. Briefly, the DCT was PCR amplified and ligated into Ca_V_1.3 via a unique XbaI site. To generate the point mutation (S1883A) in the Ca_V_1.3_S/1.4DCT_ chimeric channel, the mutation was first introduced within the Ca_V_1.4 distal C-tail peptide by QuickChange mutagenesis (Agilent) before PCR amplification and insertion into the Ca_V_1.3 channel backbone, using the same strategy as the wild-type Ca_V_1.3_S/1.4DCT_ chimeric channel. β_2a_-Gly_8_-CaM (in pcDNA3) was made by deleting the stop codon of rat β_2a_ and introducing a NotI site via PCR cloning. XbaI-glycine_8_-CaM-ApaI was then cloned into the XbaI site^42^. This construct was used for experiments in Fig. 1 and Supplementary Fig. 8; β_2a_-Gly_32_-CaM was made by extending the number of glycine residues in the linker from 8 to 32, using similar strategies^43^ and was used in experiments for Figs 3d and 4; β_1b_-Gly_32_-CaM was made by swapping β_2a_ (M80545)^44^ with β_1b_ (NM_017346.1)^45^ via KpnI and NotI sites, and was used in experiments for Figs 3h and 5.

FRET constructs were fluorescent tagged (either Venus or Cerulean) using standard PCR cloning techniques^13^. Briefly, Venus^46^ and Cerulean^47^ fluorophores (a kind gift from Dr Steven Vogel at NIH) were subcloned into pcDNA3 via unique KpnI and NotI sites. The PCR-amplified channel peptides^20^ were then cloned in via unique NotI and XbaI sites. The fluorescent-tagged peptides were PCR amplified as a whole and cloned into pAdLox via KpnI and EcoRI.

To make the Venus-PKAca-P2A-PKAr2b-Cerulean construct, Cerulean was PCR amplified and cloned into pcDNA3 via XbaI/ApaI and then PKAr2b (Addgene: pDONR223-PRKAr2b) was PCR amplified (the P2A sequence, FQGPGATNFSLLKQAGDVEENPGPSLSK, was fused 5′ of PRAr2b by this PCR amplification) and cloned via NotI/XbaI. PKAca (Addgene: pDONR223-PRKACA) was first cloned into Venus-C1 (made by replacing EGFP by Venus in pEGFP-C1)^33^. The Venus-PKAca was then PCR amplified and cloned into pcDNA3 containing P2A-PKAr2b-Cerulean via KpnI/NotI. The PKAca-P2A-PKAr2b (no fluorescent tag) was PCR amplified using Venus-PKAca-P2A-PKAr2b-Cerulean as a template and inserted into pcDNA3 via KpnI and XbaI. See Supplementary Table 3 for primer sequences.

### Adenovirus production

Adenoviral vectors were generated using the Cre-Lox recombination system^48^. pAdLox plasmids encoding the FRET binding partners were co-transfected with Ψ5 vector into Cre8 cells^49^ (a kind gift from Dr David C. Johns), allowing expression and packaging of the viral particles. After three rounds of expansion in Cre8 cells, a final viral amplification was done in HEK293 cells (ATCC). The viral particles were collected and purified by a standard CsCl gradient protocol. The purified viral stock had a titre of at least 10^12^ particles per microlitre.

### aGPVM culture and infection

Adult guinea pig ventricular myocytes (aGPVMs) were isolated from adult Hartley guinea pigs (either gender, 3–4 weeks old, weight 250–350 g) via Langendorff perfusion^30^. All procedures performed were in compliance with the Johns Hopkins School of Medicine Institutional Animal Care and Use Committee guidelines. Following isolation, myocytes were plated on laminin-coated glass coverslips in M199 media containing 20% fetal bovine serum (FBS). After 1 h, media was exchanged with 0% FBS M199 media and adenoviruses encoding FRET binding partners were added. Eight to 12 h later, the myocytes were washed once with warmed PBS and the media was replaced with fresh 0% FBS media. aGPVMs were imaged within 24 h of isolation.

### Transfection of HEK293 cells

For electrophysiology experiments, HEK293 cells (ATCC) were cultured on 10-cm plates and channels were transiently transfected by a calcium phosphate protocol^12^. Briefly, DNA was combined with 125 mM CaCl_2_ in a HEPES buffered saline solution and allowed to precipitate for 15 min. The DNA precipitate solution was then slowly added to cells and allowed to incubate for 3–5 h before solution exchange. We applied 4–16 μg of plasmid DNA encoding the desired α_1_-subunit, as well as 6–8 μg of β and 5–8 μg of rat α_2_δ (NM012919.2)^50^ subunits along with 3 μg of SV40 T antigen. For forskolin incubation experiments, β_2a_ without a glycine-linked CaM was used. In addition, 4 μg Venus-PKAca-P2A-PKAr2B-Cerulean and 1 μg PKAr2b-Cerulean were co-transfected. For dynamic forskolin wash-on experiments, a β-subunit (β_2a_ for Ca_V_1.3 and 1.4; β_1b_ for Ca_V_1.2) with glycine-linked CaM was used. In addition, 4 μg Venus-PKAca-P2A-PKAr2b-Cerulean, 1 μg PKAr2b-Cerulean and 3 μg AKAP79 (M90359.1) were co-transfected. AKAP79 is known to bind to PKA and participate in PKA regulation in primary cells^26^ and is included specifically to prevent dialysis of the PKA in whole-cell experiments. In general, β_2a_ was chosen to minimize the confounding effects of voltage-dependent inactivation, thus maximizing the dynamic range of CDI. For Ca_V_1.2 channels, β_1b_ was used to closely recapitulate the behaviour of the native Ca_V_1.2 channel expressed in cardiac cells. By decreasing the dynamic range of observable CDI, this perturbation also increased the stringency of the test and allowed for comparison with native PKA modulation of Ca_V_1.2 channels (Supplementary Fig. 5).

For microscope-based FRET assays, HEK293 cells cultured on 3.5-cm culture dishes with integral No. 0 glass coverslip bottoms (In Vitro Scientific) were transiently transfected with polyethylenimine reagent (Polysciences). For flow-cytometer-based FRET assays, HEK293T cells cultured on six-well plates were transiently transfected with polyethylenimine. All of constructs were driven by a cytomegalovirus promoter. Experiments were performed 1–2 days following transfection.

### Whole-cell electrophysiology recording

Whole-cell recordings were obtained at either room temperature or 30 °C as described, using a TC-10 heating and cooling temperature controller (Dagon Corporation) and Axopatch 200A amplifier (Axon Instruments). Electrodes were pulled from borosilicate glass capillaries (Word Precision Instruments), with 1–3 MΩ resistances, which were in turn compensated for series resistance by >60%. Currents were low-pass filtered at 2 kHz before digital acquisition at five times the frequency. A P/8 leak-subtraction protocol was used. The internal solution contained (in mM): CsMeSO_3_, 114; CsCl, 5; MgATP, 4; HEPES pH 7.4, 10; and BAPTA (1,2-bis(*o*-aminophenoxy)ethane- *N*,*N*,*N'*,*N'*-tetraacetic acid), 10; at 295 mOsm adjusted with CsMeSO_3_. The bath solution was (in mM): TEA-MeSO_3_, 102; HEPES pH 7.4, 10; CaCl_2_ or BaCl_2_, 40; at 305 mOsm adjusted with TEA-MeSO_3_.

### Microscope-based FRET optical imaging

FRET two-hybrid experiments were performed on an inverted microscope^13^,^51^. Briefly, total fluorescence from single cells, as isolated via pinhole in the image plane, was quantified by a photo-multiplier tube. The bath Tyrode's solution was (in mM): NaCl, 138; KCl, 4; MgCl_2_, 1; HEPES pH 7.4, 10; CaCl_2_, 2; at 305 mOsm adjusted with glucose. Filter cubes (excitation, dichroic, emission and company): CFP (D440/20X, 455DCLP, D480/30M, Chroma); YFP (500AF25, 525DRLP, 530ALP, Omega Optical); and FRET (D436/20X, 455DCLP, D535/30M, Chroma). The YFP signal was measured directly by excitation/emission through the YFP cube, thus was used as a gage for nonspecific fluorophore quenching or movement artefacts. FRET changes were determined via the CFP and FRET cubes^13^,^51^. Background fluorescent signals were measured from cells without expression of the fluorophores, which was particularly important for experiments in aGPVMs due to higher autofluorescence. In HEK cells, concentration-dependent spurious FRET was subtracted from the raw data before binding-curve analysis^13^,^51^. Cerulean and Venus were used as the donor and acceptor fluorescent proteins instead of cyan fluorescent protein (CFP) and yellow fluorescent protein (YFP), as their optical properties are better for this application.

Acceptor-centric measurements of FRET were obtained with the 3^3^-FRET algorithm^13^,^51^, which expresses the effective FRET efficiency (*E*_eff_) and FR as:


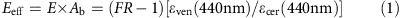


where 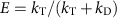 is the FRET efficiency of a donor–acceptor pair, 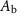 is the fraction of acceptor molecules bound by a donor. 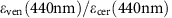 is the approximate molar extinction coefficients of Cerulean and Venus, which was measured as 0.08 on our setup.

Analysis was done using a previously described binding model^13^,^51^ as follows. A 1:1 ligand-binding model is assumed to determine two parameters, FR_max_ and *K*_d,EFF_. FR_max_ is the maximum FR that occurs when all acceptor-tagged molecules are bound; hence, FR_max_ depends only on inter-fluorophore geometry. The second parameter *K*_d,EFF_, the effective dissociation constant, furnishes the relative dissociation constant for the binding reaction, with conversion factors to actual *K*_d_ determined by optical characteristics of our microscope system. *D*_free_ is the relative concentration of free donor molecules. All parameters determined for each construct in this study are listed in Supplementary Table 1.

### Flow-cytometer-based FRET optical imaging

FRET two-hybrid experiments were performed on an Attune Acoustic Focusing Cytometer (Life Technologies)^33^. Cells were trypsinized and resuspended in Tyrode's solution before loading onto the flow cytometer. Optical bandpass filters used for CFP, YFP and FRET were 450/40, 530/30 and 522/31 nm, respectively. Cells were illuminated for 40 μs at each excitation wavelength. The above algorithm (equation (1))^13^,^51^ was used to calculate acceptor-centric measures. In total, signals from 1,000–10,000 cells were recorded; however, only 30–50 randomly selected cells were displayed, to improve clarity of the figure. Flow-cytometer-based FRET correlated precisely with the microscope-based FRET^33^, but afforded much greater throughput. Parameters pertaining to the flow-cytometer-based FRET assay are listed in Supplementary Table 2.

### Mass spectroscopy

Double polyhistidine (His_8_)-tagged Cerulean-ICDI_1.4_ peptides were co-transfected with PKA holoenzyme in HEK293T cells. Cells were incubated in 50 μM forskolin in a 37 °C incubator for 1 h before collecting with a lysis buffer, containing (in mM): KCl, 20; K-gluconate, 120; MgCl_2_, 2; EGTA, 0.2; HEPES pH 7.4; Na_2_-ATP, 2; dithiothreitol, 10; cAMP, 0.1. Protease inhibitor cocktail (Roche, complete, EGTA-free) and phosphatase inhibitor cocktail 2 and 3 (Sigma-Aldrich) were added into the lysis buffer according to the standard protocol. His-tagged Cerulean-ICDI_1.4_ peptides were purified by Ni-NTA agarose (Qiagen) and verified by SDS–PAGE using mini-PROTEAN precast gel (Bio-Rad). The band containing the ICDI_1.4_ peptide (Supplementary Fig. 7a) was cut from the gel then sent to the Johns Hopkins Mass Spectroscopy and Proteomics Facility, where the peptides were in-gel proteolysed with trypsin and analysed on nano-liquid chromatography electrospray ionization/tandem mass spectrometry Orbi-trap Velos in high resolution Fourier transform mode. Tandem MS2 mass spectra were processed by Proteome Discoverer (v1.4 Thermo Fisher Scientific) in three ways, using three Nodes: common, Xtract (spectra were extracted, charge state deconvoluted and deisotoped using Xtract option) and MS2 Processor. The tandem mass spectra were searched against the RefSeq2014 Human database filtered at a 1% false dicovery rate using PEAKS 7.0 (Bioinformatics Solutions Inc.). Variable modifications by M oxidation, NQ deamidation and STY phosphorylation were searched for with a precursor ion tolerance of 10 p.p.m. and a fragment ion tolerance of 0.02 Da.

Two peptides (peptide 1: GSADSLVEAVLISEGLGLFAR and peptide 2: LTLDEMDNAASDLLAQGTSSLYSDEESILSR) from Ca_V_1.4 were identified, which covered 52% of the total 100 amino acids in ICDI_1.4_ (Supplementary Fig. 6d) and 11 of the 14 potential phosphorylation sites in this region. Three potential sites, T1872, S1874 and S1877, were not covered due to the limitation of trypsin, but they are not in the core region for IQ interaction^28^. Peptide 1 was identified nine times, among which it carried a phosphate group six times. The peaks from double and triple charged peptides detected in the first MS showed a molecular weight that was 79.97 Da higher than that expected without a phosphate group, verifying phosphorylation. The data predicted that either S1883 or S1886 could be the site of phosphorylation. However, the existence of b_4_, b_4_(–98) and y_17_ in the spectra in (Fig. 6a and Supplementary Fig. 7b) strongly suggested that the phosphorylation site is S1883. In addition, pkaPS predictions and mutagenesis (Fig. 6 and Supplementary Fig. 6) strongly support that the PKA phosphorylation site is S1883. Peptide 2 was identified 12 times and carried a phosphate group at S1942 in only 1 trial. This potential phosphorylation site, however, did not have any effect on the interaction between IQ_1.3_ and ICDI_1.4_ in our FRET binding assay (Supplementary Fig. 6c,d).

### Creating a robust and controllable PKA system in HEK293 cells

Although PKA regulation of the IQ_1.3_/ICDI_1.4_ interaction was easily observed in aGPVMs (Supplementary Fig. 3b,c), the same regulation was absent in HEK293 cells (Supplementary Fig. 1a), until the PKA holoenzyme (both the regulatory and catalytic subunits) was overexpressed (Supplementary Fig. 1b). Based on this evidence, we hypothesized that the expression level of PKA is lower in HEK293 cells than aGPVMs. Thus, the PKA holoenzyme was overexpressed in most of our experiments in HEK293 cells.

The delicately balanced expression of regulatory and catalytic subunits of PKA in native systems cannot be easily achieved by standard heterologous expression, as co-transfection of both subunits results in mixed basal PKA activity. To mimic the endogenous expression of native systems and guarantee low basal PKA activity, the overexpressed regulatory subunits would have to slightly outnumber the overexpressed catalytic subunit. To achieve this, we linked the catalytic and regulatory subunits by a viral-based 2A sequence from porcine tescho virus (P2A) optimized and characterized in a previous study^52^. The P2A sequence causes a self-hydrolysis reaction at the translation level, resulting in complete dissociation and close to 1:1 stoichiometry of the linked subunits. A small amount of extra regulatory subunit (1 μg per 4 μg holoenzyme) was co-expressed with this P2A construct to guarantee that the regulatory subunit always slightly outnumbered the catalytic subunit in each cell.

To test our system, we used the FRET-based PKA activity sensor AKAR4 (ref. 53). First, we expressed AKAR4 in HEK293 cells and measured the FRET efficiency (*E*_eff_) of each cell by a flow cytometry assay. At this endogenous PKA expression level, most cells exhibited low PKA activity, as illustrated by the low *E*_eff_ of AKAR4, whereas a small percentage of cells showed high basal PKA activity (Supplementary Fig. 9). When PKA holoenzyme was expressed as described above, every cell had minimal basal PKA activity. In addition, when the cAMP level was elevated by adding 50 μM forskolin, PKA activity was saturated in all cells, indicated by the high *E*_eff_ of AKAR4 (Supplementary Fig. 9).

Finally, recording for forskolin wash-on experiments was done at a temperature of 30 °C, which was determined to be a necessary condition for robust PKA modulation (Supplementary Fig. 4). Of note, increasing the temperature from 23 °C to 30 °C significantly increased the extent of PKA modulation achieved by a 30-min incubation with forskolin and increasing the temperature from 30 °C to 37 °C further enhanced modulation (Supplementary Fig. 4). Thus, the PKA process for ICDI_1.4_ interaction with the channel IQ domain is much slower than many traditional PKA-mediated processes. The slow onset of PKA modulation fits well with the time course of channel regulation achieved by enriching CaM at the membrane via a rapamycin-based system^14^ and may represent a time course that is physiologically relevant in the visual system, where dopamine drives PKA slowly according to the circadian rhythm^10^,^54^.

### Data availability

The data that support the findings of this study are available from the corresponding author upon reasonable request.

## Additional information

**How to cite this article:** Sang, L. *et al*. Protein kinase A modulation of Ca_V_1.4 calcium channels. *Nat. Commun.* 7:12239 doi: 10.1038/ncomms12239 (2016).

## Supplementary Material

Supplementary Information Supplementary Figures 1-9, Supplementary Tables 1-3 and Supplementary References.
