# Supplementary material for: Protein kinase A modulation of CaV1.4 calcium channels
Source: Nat Commun. 2016 Jul 26;7:12239. doi: 10.1038/ncomms12239 (PMC4963476; doi:10.1038/ncomms12239)
Supplement: Supplementary Information — Supplementary Figures 1-9, Supplementary Tables 1-3 and Supplementary References. [file ncomms12239-s1.pdf]

Supplementary Information

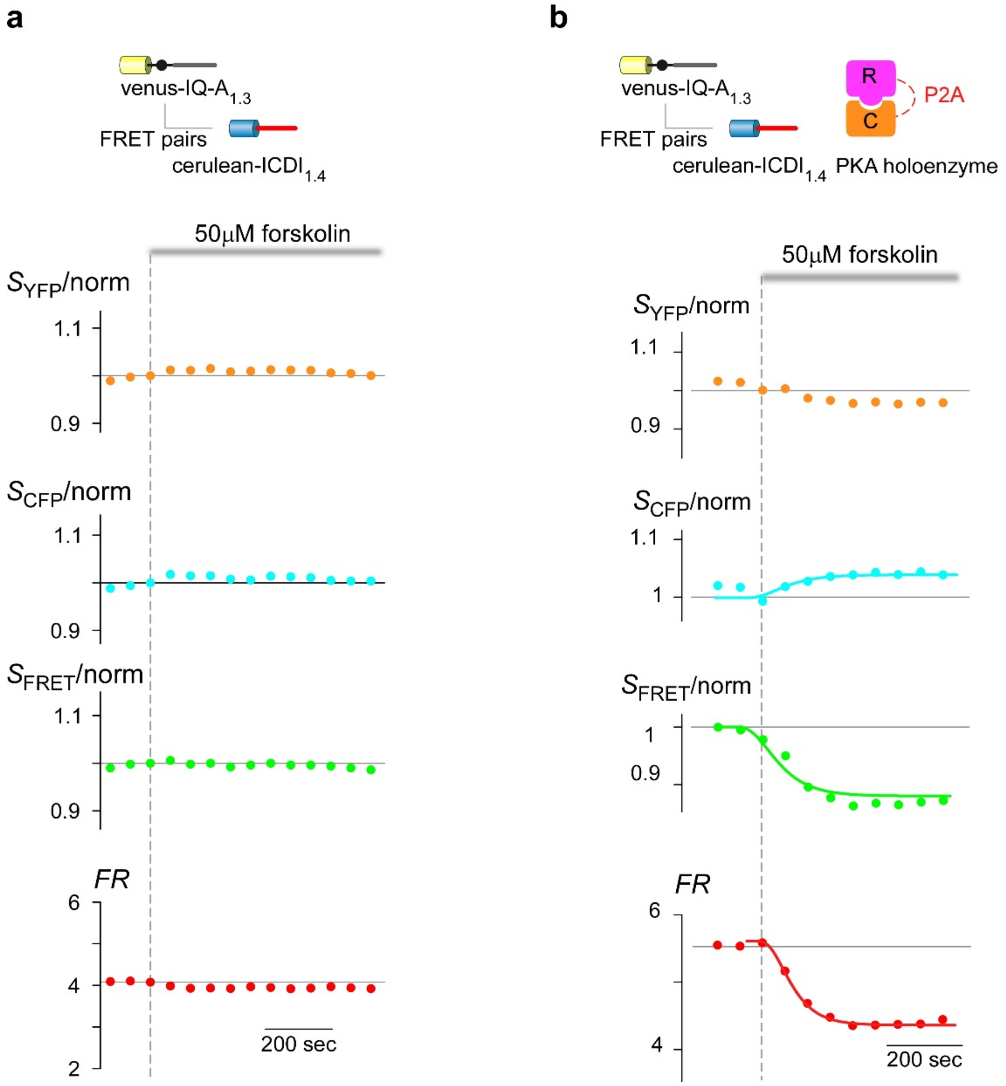

Supplementary Figure 1 | Forskolin regulates the interaction between $IQ_{1.3}$ and $ICDI_{1.4}$ in HEK293 cells only when PKA holoenzyme is co-expressed. (**a**) Time course of normalized fluorescent signals from YFP channel (S_{YFP}), CFP channel (S_{CFP}), FRET channel (S_{FRET}) and calculated FRET ratio (*FR*) from an exemplar HEK293 cell expressing Venus-IQ-$A_{1.3}$ and Cerulean-$ICDI_{1.4}$ peptides. 50 μM forskolin applied as indicated. (**b**) Time course of normalized fluorescent signals and calculated *FR* from an exemplar HEK293 cell expressing Venus-IQ-$A_{1.3}$, Cerulean-$ICDI_{1.4}$ peptides, and PKA holoenzyme (see Methods).

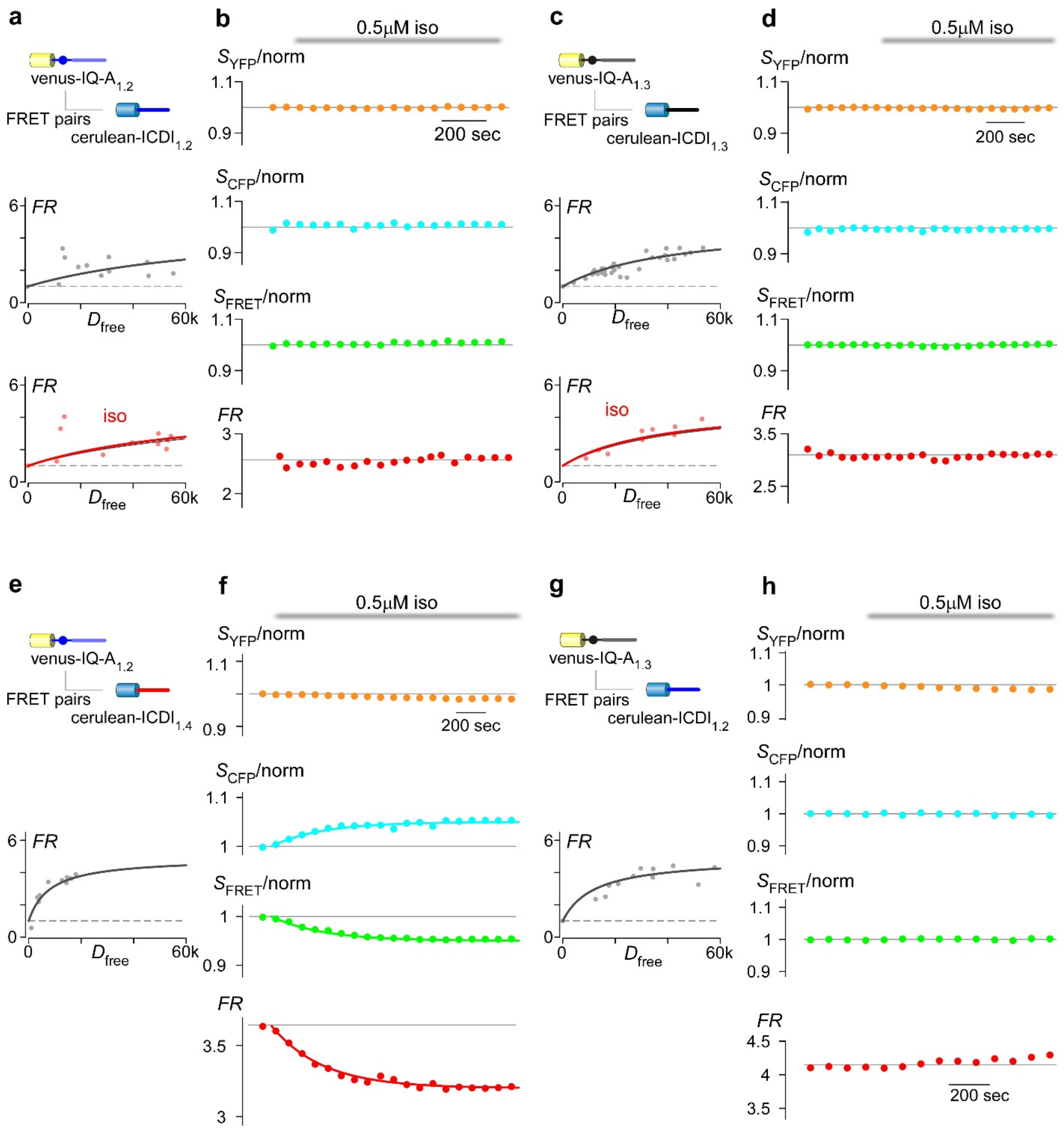
a
venus-IQ-A1.2
FRET pairs
cerulean-ICDI1.2
FR
iso
Dfree
60k
b
0.5μM iso
SYFP/norm
200 sec
SCFP/norm
SFRET/norm
FR
c
venus-IQ-A1.3
FRET pairs
cerulean-ICDI1.3
d
e
venus-IQ-A1.2
FRET pairs
cerulean-ICDI1.4
f
g
venus-IQ-A1.3
FRET pairs
cerulean-ICDI1.2
h

Supplementary Figure 2 | PKA regulation of IQ and ICDI interactions in aGPMVs. (**a**) FRET binding assays between Venus-IQ-$A_{1.2}$ and Cerulean-$ICDI_{1.2}$ peptides, without (gray) or with (red) application of isoproterenol (iso). The grey curve in the middle panel is replicated in the lower panel, overlapping the red curve. (**b**) Kinetics of normalized fluorescent signals from the YFP channel (S_{YFP}), CFP channel (S_{CFP}), FRET channel (S_{FRET}) and calculated FRET ratio (*FR*) from an exemplar aGPVM expressing Venus-IQ-$A_{1.2}$ and Cerulean-$ICDI_{1.2}$. Isoproterenol was applied as indicated, without any effect. Fluorescent signals were normalized to baseline. (**c**) FRET binding assays between Venus-IQ-$A_{1.3}$ and Cerulean-$ICDI_{1.3}$ peptides, without (gray) or with (red) isoproterenol. (**d**) Lack of response to isoproterenol by an exemplar aGPVM expressing Venus-IQ-$A_{1.3}$ and Cerulean-$ICDI_{1.3}$ peptides. (**e**) FRET binding curve indicating robust interaction between Venus-IQ-$A_{1.2}$ and Cerulean-$ICDI_{1.4}$ peptides. (**f**) Isoproterenol causes a significant decrease in *FR* within an exemplar aGPVM expressing Venus-IQ-$A_{1.2}$ and Cerulean-$ICDI_{1.4}$ peptides. (**g**) FRET binding assay between Venus-IQ-$A_{1.3}$ and Cerulean-$ICDI_{1.2}$ peptides. (**h**) Lack of response to isoproterenol in an exemplar aGPVM expressing Venus-IQ-$A_{1.3}$ and Cerulean-$ICDI_{1.2}$ peptides.

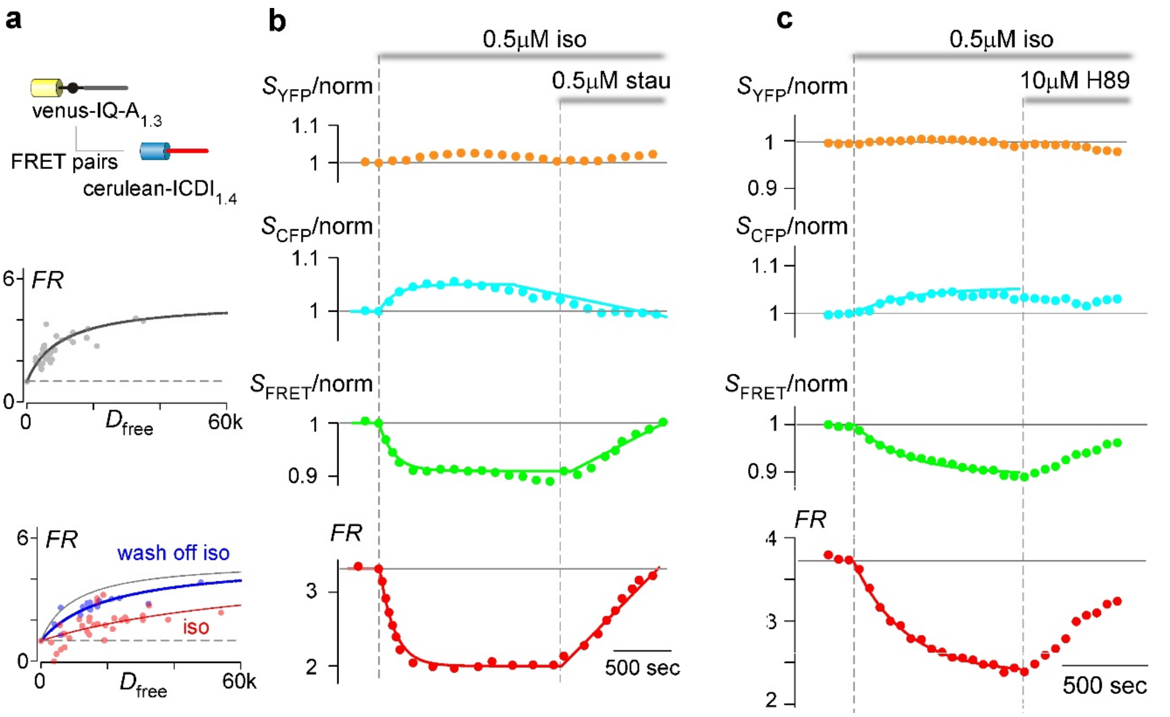

Supplementary Figure 3 | PKA regulates the interaction between $IQ_{1.3}$ and $ICDI_{1.4}$ in aGPVMs. (**a**) FRET binding curve for Venus-IQ-$A_{1.3}$ paired with Cerulean-$ICDI_{1.4}$ (gray). 0.5 µM isoproterenol (red) decreases the relative binding affinity and washoff of the isoproterenol reverses this effect (blue). Each point indicates a single cell. The control binding curve is replicated as the gray curve in the lower panel. *FR*, FRET ratio; D_{free}, relative concentration of unbound Cerulean-tagged $ICDI_{1.4}$. (**b**) Kinetics of normalized fluorescent signals from YFP channel (S_{YFP}), CFP channel (S_{CFP}), FRET channel (S_{FRET}) and calculated *FR* from an exemplar aGPVM expressing Venus-IQ-$A_{1.3}$ and Cerulean-$ICDI_{1.4}$, with 0.5 µM isoproterenol and 0.5 µM staurosporine added as indicated. Fluorescent signals were normalized to baseline here and throughout. (**c**) Kinetics of normalized fluorescent signals and *FR* from an exemplar aGPVM expressing Venus-IQ-$A_{1.3}$ and Cerulean-$ICDI_{1.4}$, with 0.5 µM isoproterenol and 10 µM H89 added as indicated. For this data set, a movement artifact was noted in the raw YFP signal and was divided out of the final data set.

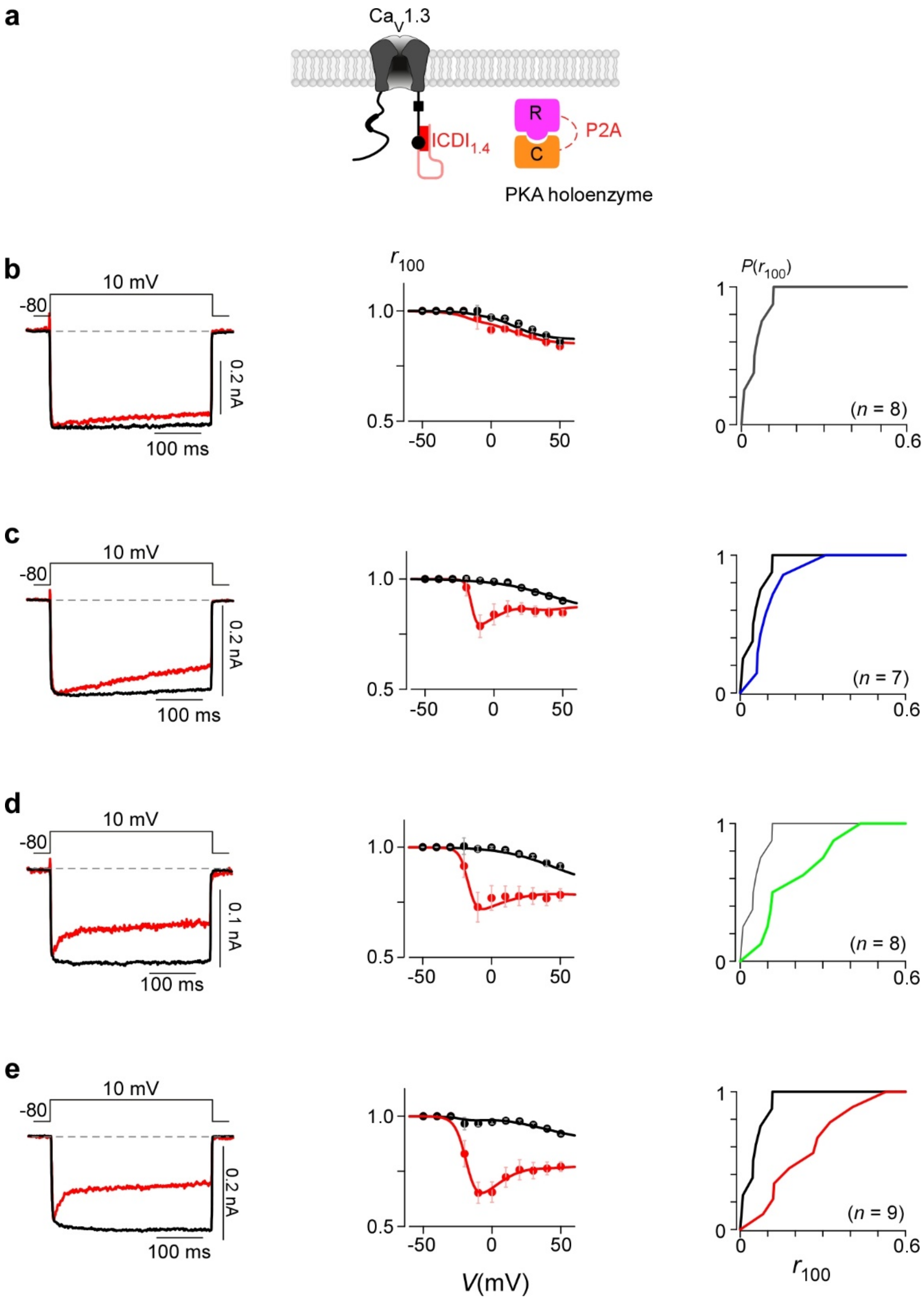

a
CaV1.3
ICDI1.4
R
C
P2A
PKA holoenzyme
b
c
d
e
10 mV
-80
0.2 nA
0.1 nA
100 ms
r100
1.0
0.5
-50
0
50
V(mV)
P(r100)
1
0
0.6
(n = 8)
(n = 7)
(n = 8)
(n = 9)
r100

Supplementary Figure 4 | Temperature dependence of PKA modulation of $Ca_V1.3_{S/1.4DCT}$ (**a**) Schematic of HEK293 cells co-expressing $Ca_V1.3_{S/1.4DCT}$ channels, PKA holoenzyme (catalytic subunit, C and regulatory subunit, R, linked by a P2A sequence at the DNA level, see Methods). (**b**) Minimal CDI at basal PKA levels. (Left) Exemplar of whole-cell current illustrates a lack of CDI, as seen by the minimal difference in decay of Ca^{2+} (red) versus Ba^{2+} (black) current. Vertical scale bar pertains to Ca^{2+} current; Ba^{2+} current scaled downwards ~3× to facilitate comparison of decay kinetics, here and throughout. (Middle) Population data confirmations minimal CDI. The fraction of peak current remaining after 100-ms (r_{100}) is plotted versus step voltage (V), for Ba^{2+} (black) and Ca^{2+} (red) currents (mean ± SEM). (Right) The tight cumulative distribution of r_{100} across all cells also indicates minimal CDI. (**c**) CDI emerges after incubation in 50 μM forskolin at 23°C for 30 minutes. (**d**) Incubation with 50 μM forskolin for 30 min at 30°C increases the extent of CDI. (**e**) Incubation with 50 μM forskolin for 30 min at 37°C further increases the extent of CDI.

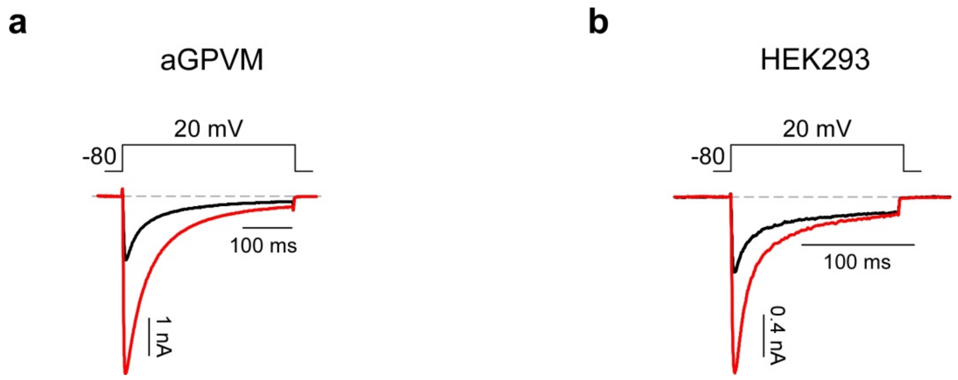

Supplementary Figure 5 | Comparison of the native and synthetic regulation of $Ca_V1.2$. (**a**) Ca^{2+} current elicited by step depolarization from -80mV to 20mV from an aGPVM, before (black) and after (red) application of 0.5μM isoproterenol. (**b**) Ca^{2+} current from a HEK293 cell expressing $Ca_V1.2$, $ICDI_{1.4}$ peptide and PKA holoenzyme (Fig. 5), elicited by step depolarization from -80mV to 20mV, before (black) and after (red) application of 50 μM forskolin.

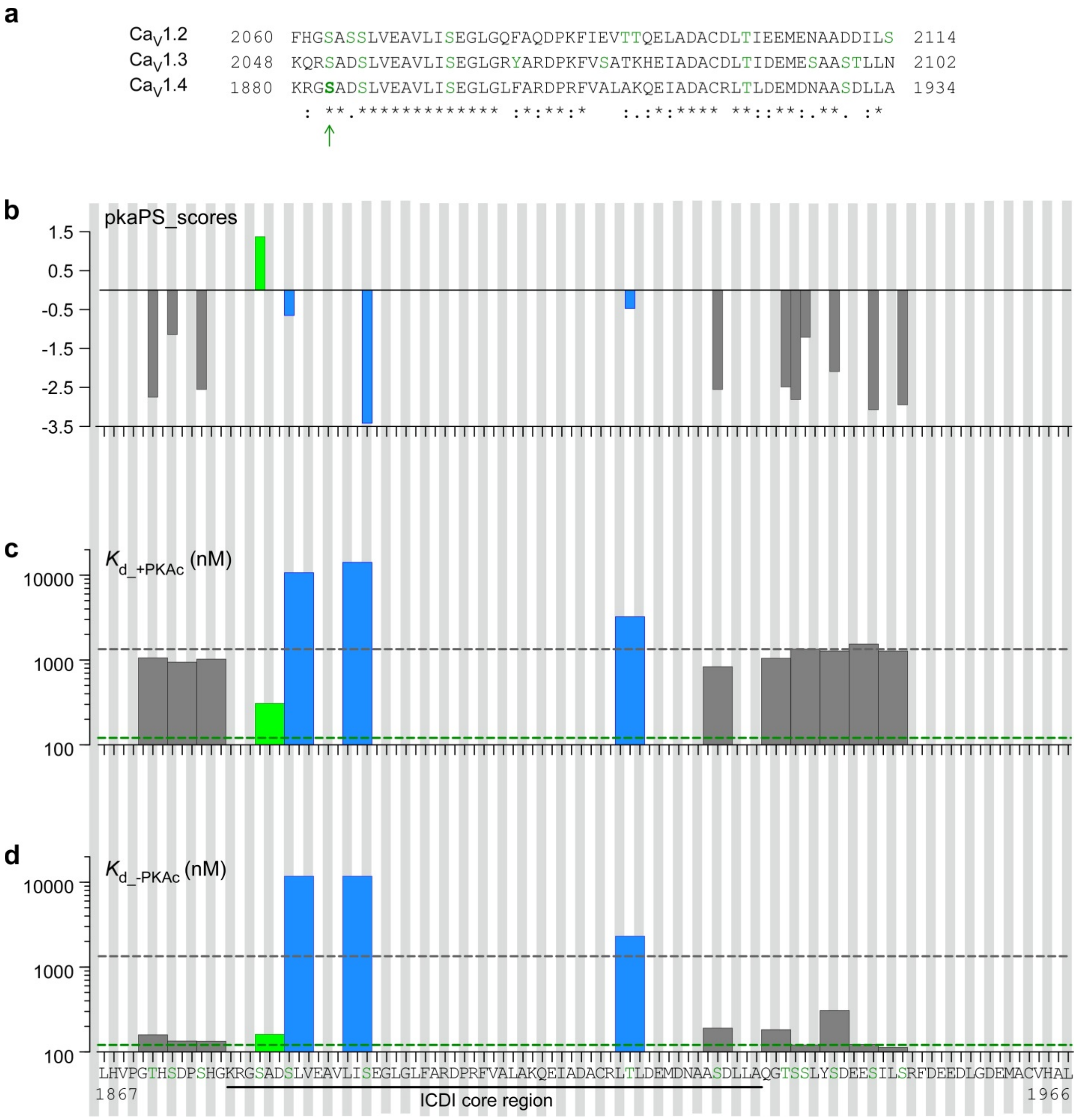
a
CaV1.2 2060 FHGSASSLVEAVLISEGLGQFAQDPKFIEVTTQELADACDLTIEEMENAADDILS 2114
CaV1.3 2048 KQRSADSLVEAVLISEGLGRYARDPKFVSATKHEIADACDLTIDEMESAASTLLN 2102
CaV1.4 1880 KRGSADSLVEAVLISEGLGLFARDPRFVALAKQEIADACRLTLDEMDNAASDLLA 1934
b
pkaPS_scores
1.5
0.5
-0.5
-1.5
-2.5
-3.5
c
Kd_+PKAc (nM)
10000
1000
100
d
Kd_-PKAc (nM)
10000
1000
100
LHVPGTHSDPSHGKRGSADSLVEAVLISEGLGLFARDPRFVALAKQEIADACRLTLDEMDNAASDLLAQGTSSLYSDEESILSRFDEEDLGDEMACVHAL
1867
1966
ICDI core region

Supplementary Figure 6 | Identifying the PKA phosphorylation site on Ca_V1.4. (**a**) Sequence alignment of the core region of ICDI modules (identified by alanine scanning in previous studies[1]) from Ca_V1.2, 1.3 and 1.4, with consensus symbols labeled underneath. Potential phosphorylation sites (STY) are colored green. The arrow indicates S1883. (**b**) pkaPS scores of potential phosphorylation sites in $ICDI_{1.4}$. The corresponding sequence is displayed at the bottom on the x-axis in **d**. S1883 (green) has the highest score. (**c**) Dissociation constants (K_{d_+PKAc}) measured by FRET two-hybrid assays between Venus-IQ-$A_{1.3}$ and cereulan-$ICDI_{1.4}$ peptides in the presence of overexpressed PKAc. Every three contiguous residues within $ICDI_{1.4}$ (corresponding to the bottom sequence in **d**) were systematically substituted by alanines[1]. The green dashed line marks the dissociation constant between Venus-IQ-$A_{1.3}$ peptide and wild-type cereulan-$ICDI_{1.4}$ peptides without overexpressing PKAc. The gray dashed line marks the dissociation constant between Venus-IQ-$A_{1.3}$ and wild-type cereulan-$ICDI_{1.4}$ peptides with overexpression of PKAc. With SAD1883AAA mutations (green), overexpressing PKAc fails to increase the dissociation constant of the peptides (compare to **d**, green bar) indicating that S1883 is likely the PKA phosphorylation site. (**d**) Dissociation constants (K_{d_-PKAc}) measured by FRET two-hybrid assays between Venus-IQ-$A_{1.3}$ and cereulan-$ICDI_{1.4}$ peptides without overexpressed PKAc. The green and gray dashed lines are the same as in **c**. The corresponding $ICDI_{1.4}$ sequence is labeled under the x-axis. The core region[1] for IQ interaction is marked by the bar below the sequence. Mutations in three constructs (blue bars) reduced the dissociation constants of IQ/ICDI interaction even at basal PKA levels.

a

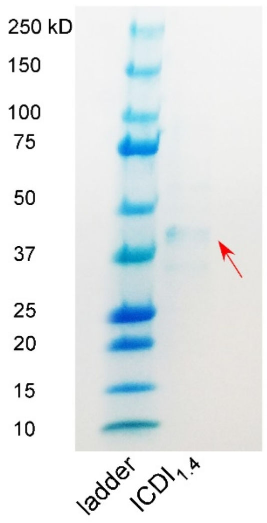

b

#	b	b-H2O	b-NH3	b (2+)	Seq	y	y-H2O	y-NH3	y (2+)	#
1	58.03	40.02	41.00	29.51	G					21
2	225.03	207.02	208.00	113.01	S(+79.97)	2127.07	2109.06	2110.05	1064.04	20
3	296.06	278.05	279.04	148.53	A	1960.07	1942.06	1943.05	980.54	19
4	411.09	393.08	394.06	206.05	D	1889.04	1871.03	1872.01	945.02	18
5	498.12	480.11	481.10	249.56	S	1774.01	1756.00	1756.98	887.51	17
6	611.21	593.20	594.18	306.10	L	1686.98	1668.97	1669.95	843.99	16
7	710.29	692.27	693.25	355.64	V	1573.89	1555.88	1556.87	787.45	15
8	839.32	821.31	822.29	420.16	E	1474.82	1456.81	1457.80	737.91	14
9	910.36	892.35	893.33	455.68	A	1345.78	1327.77	1328.76	673.39	13
10	1009.42	991.41	992.40	505.21	V	1274.74	1256.74	1257.72	637.87	12
11	1122.51	1104.50	1105.48	561.75	L	1175.68	1157.66	1158.67	588.34	11
12	1235.59	1217.58	1218.57	618.30	I	1062.59	1044.59	1045.57	531.80	10
13	1322.62	1304.61	1305.60	661.81	S	949.51	931.49	932.48	475.26	9
14	1451.67	1433.66	1434.64	726.33	E	862.48	844.47	845.45	431.74	8
15	1508.69	1490.68	1491.66	754.84	G	733.43	715.42	716.41	367.22	7
16	1621.77	1603.76	1604.75	811.39	L	676.41	658.40	659.39	338.71	6
17	1678.79	1660.78	1661.77	839.90	G	563.33	545.32	546.30	282.18	5
18	1791.88	1773.87	1774.85	896.44	L	506.31	488.30	489.28	253.65	4
19	1938.95	1920.94	1921.92	969.97	F	393.22	375.21	376.20	197.11	3
20	2009.98	1991.97	1992.96	1005.49	A	246.16	228.15	229.12	123.58	2
21					R	175.12	157.11	158.09	88.06	1

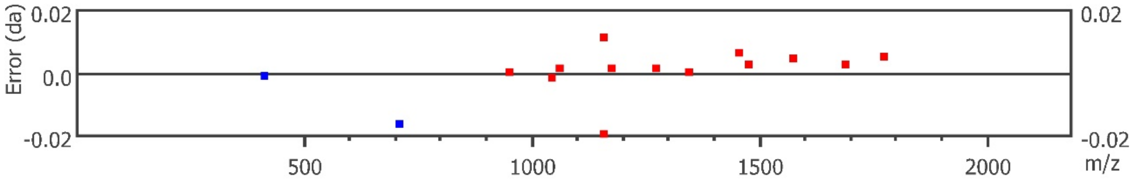

Supplementary Figure 7 | Additional information for mass spectroscopy experiments. (**a**) Coomassie blue stained SDS-PAGE gel loaded with purified double-histidine-labeled Cerulean-$ICDI_{1.4}$ peptides (expected molecular weight: 40.8kD). The red arrow indicates the band analyzed by mass spectroscopy. (**b**) Ion table and error map of the spectra in Fig. 6a.

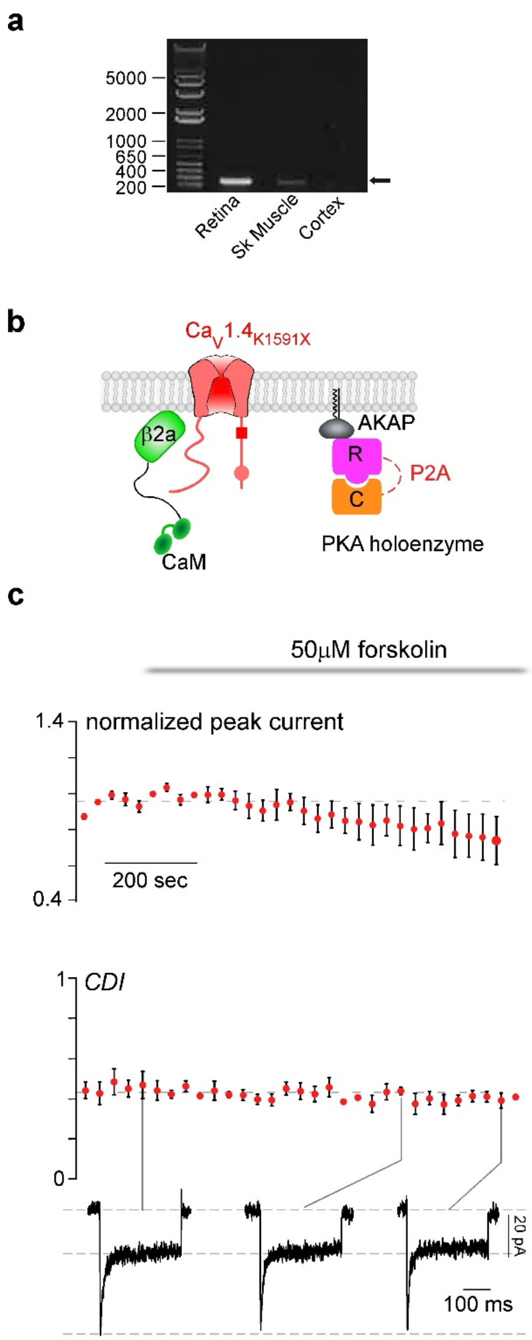

Supplementary Figure 8 | PKA regulation of Ca$_V$1.4 is disrupted in congenital stationary night blindness. (**a**) RT-PCR results demonstrating high expression of ICDI$_{1.4}$ in adult guinea pig retina, lower expression in skeletal muscle and no expression in cortex, matching the known expression pattern of Ca$_V$1.4 channels. (**b**) Schematic of co-expression of the congenital stationary night blindness mutant channel Ca$_V$1.4$_{K1591X}$ with PKA holoenzyme, AKAP79 and β$_{2a}$ tethered CaM which allowed longer whole cell patching without loss of PKA or CaM. (**c**) Time course of normalized peak current (red) and steady state current (gray) in response to the addition of 50 μM forskolin, evoked every 30 seconds by depolarizations to 20 mV (top). No increase in current amplitude was seen as compared to a significant increase displayed by WT Ca$_V$1.4 channels (Fig. 1d). CDI was larger at baseline as compared to WT channels, and no change was elicited by forskolin washon (middle). Currents were normalized to baseline. CDI was measured as 1 - r_{100}. All error bars indicate +/- SEM (n = 3 cells). Corresponding current waveforms are displayed below.

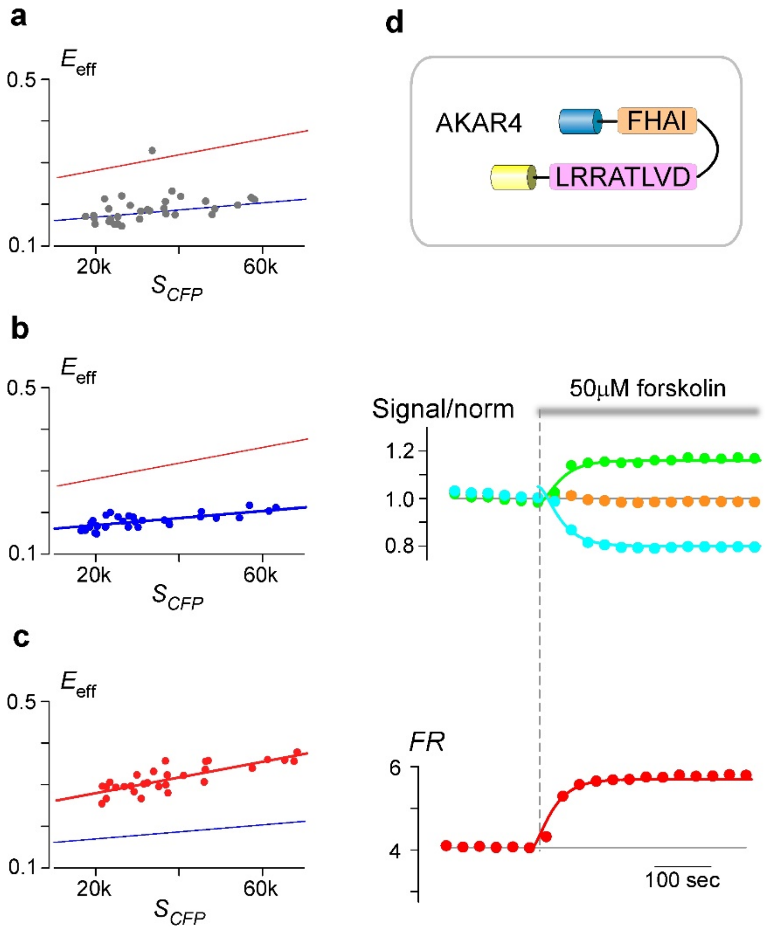

Supplementary Figure 9 | Co-expressing PKA holoenzyme ensures robust and controllable PKA activity. (**a**) FRET efficiency (E_{eff}) measured for HEK293 cells expressing the PKA sensor AKAR4[2]. Each point indicates a single cell. The blue line represents low PKA activity, and the red line indicates high PKA activity. (**b**) E_{eff} measured for HEK293 cells expressing AKAR4 and PKA holoenzyme, without forskolin treatment. (**c**) E_{eff} measured for forskolin treated HEK293 cells expressing AKAR4 and PKA holoenzyme, recorded after incubation in 50μM forskolin for 10 minutes. (**d**) An exemplar recording demonstrating the kinetics of the AKAR response in HEK 293 cells. YFP signal: yellow; CFP signal: cyan; FRET signal: green; *FR*: red.

Venus-IQA	Cerulean-ICDI	PKA†	cell type	K_{d_EFF}	FR_{max}	E_{max}‡	Figure
$Ca_V1.4$	$Ca_V1.4$	-	aGPVM	8,000	4.9	0.23	2c, grey
$Ca_V1.4$	$Ca_V1.4$	+	aGPVM	50,000	4.9	0.23	2c, red
$Ca_V1.4$	$Ca_V1.4$	-	HEK293	3,000	5.7	0.3	2e, grey
$Ca_V1.4$	$Ca_V1.4$	+	HEK293	15,000	5.7	0.3	2e, red
$Ca_V1.3$	$Ca_V1.3$	-	aGPVM	42,000	4.9	0.23	Supp 2c, grey
$Ca_V1.3$	$Ca_V1.3$	+	aGPVM	38,000	4.9	0.23	Supp 2c, red
$Ca_V1.3$	$Ca_V1.3$	-	HEK293	28,000	5.7	0.3	3b, grey
$Ca_V1.3$	$Ca_V1.3$	+	HEK293	21,000	5.7	0.3	3b, red
$Ca_V1.2$	$Ca_V1.2$	-	aGPVM	80,000	4.9	0.23	Supp 2a, grey
$Ca_V1.2$	$Ca_V1.2$	+	aGPVM	70,000	4.9	0.23	Supp 2a, red
$Ca_V1.2$	$Ca_V1.2$	-	HEK293	18,000	5.7	0.3	3f, grey
$Ca_V1.2$	$Ca_V1.2$	+	HEK293	18,000	5.7	0.3	3f, red
$Ca_V1.3$	$Ca_V1.4$	-	aGPVM	10,000	4.9	0.23	Supp 3a, grey
$Ca_V1.3$	$Ca_V1.4$	+	aGPVM	75,000	4.9	0.23	Supp3a, red
$Ca_V1.3$	$Ca_V1.4$	-	HEK293	1,700	5.7	0.3	4b, grey
$Ca_V1.3$	$Ca_V1.4$	+	HEK293	10,000	5.7	0.3	4b, red
$Ca_V1.2$	$Ca_V1.4$	-	aGPVM	8,000	4.9	0.23	Supp 2e, grey
$Ca_V1.3$	$Ca_V1.2$	-	aGPVM	12,000	4.9	0.23	Supp 2g, grey

Supplementary Table 1 | Parameter values for microscope-based FRET experiments †PKA (+) activated by adding isoproterenol in aGPVMs or overexpressing PKAc in HEK293 cells (-) no PKA activation. ‡ $E_{\max} = (FR_{\max} - 1)\,\varepsilon_{\text{ven}}(440nm)/\varepsilon_{\text{cer}}(440nm)$, see equation (1) in methods and Supplemental Information of Erickson et al, Neuron 31, 973-985 for more details.

Venus-IQA	Cerulean-ICDI	PKA†	mutation	K_{d_EFF}	FR_{max}‡	E_{max}	Figure
$Ca_V1.3$	$Ca_V1.4$	-	wt	6,000	-	0.33	6c, grey
$Ca_V1.3$	$Ca_V1.4$	+	wt	60,000	-	0.33	6c, red
$Ca_V1.3$	$Ca_V1.4$	-	S1883A	2,600	-	0.33	6d, grey
$Ca_V1.3$	$Ca_V1.4$	+	S1883A	3,700	-	0.33	6e, red
$Ca_V1.3$	$Ca_V1.4$	-	S1886A	3,600	-	0.33	6e, grey
$Ca_V1.3$	$Ca_V1.4$	+	S1886A	76,000	-	0.33	6e, red

Supplementary Table 2 | Parameter values for flow-cytometer-based FRET experiments All experiments were performed using HEK293 cells. †PKA (+) activated by overexpressing PKAc (-) no PKA activation. ‡Values not measured for this setup.

Construct	Forward primers	Reverse primers	usage
$Ca_V1.3_{S/1.4DCT}$ (S1883A)	CATGGGAAGAGGGGCGATGCCGACAGCTTGGT	ACCAAGCTGTCGGCATCGCCCCTCTTCCCATG	QuickChange mutagenesis
β_{1b}-Gly$_{32}$-CaM (pcDNA3)	agcttGGTACCGGATCCGCCACCATGGTCCAGAAGAGCGGCATG	ttgtagAGCGGCCGCGCGGATGTAGACGCCTTGTCC	To clone in β_{1b}
Venus-PKAca-P2A-PKAr2b-Cerulean (pcDNA3)	catgcaTCTAGAGTGAGCAAGGGCGAGGAGC	agaataGGGCCCTTACTTGTACAGCTCGTCCATGCCGAG	To clone in Cerulean
Venus-PKAca-P2A-PKAr2b-Cerulean (pcDNA3)	gtcGCGGCCGCT*CAGGGCCCGGGCGCTACTAACTTCAGCCTGCTGAAGCAGGCTGGAGACGTGGAGGAGAACCCTGGACCT*AGCATCGAGATCCCGGCGG	ctccacTCTAGATGCAGTGGGTTCAACAATATCCATGTTC	To clone in P2A-PKAr2b
Venus-PKAca-P2A-PKAr2b-Cerulean (pcDNA3)	gattaGGTACC*GCCACC*ATGGTGAGCAAGGGCGAGG	caagAGCGGCCGCAAACTCAGAAAACTCCTTGCCACACTTC	To clone in Venus-PKAca
All FRET constructs In pcDNA3	gattaGGTACC*GCCACC*ATGGTGAGCAAGGGCGAGG	caagaGCGGCCGCCTTGTACAGCTCGTCCATGCC	To clone Venus or Cerulean into pcDNA3
Venus-IQ-A$_{1.3}$ (pcDNA3)	cgatGCGGCCGCTAAGACTGAAGGCAACCTGGAGCAAGCTAAT	tcagTCTAGA*TTATTA*TCTAGATTATTAATGGCCGTTTTCAGACACATGCTCAAG	To clone in IQ-A$_{1.3}$
Venus-IQ-A$_{1.4}$ (pcDNA3)	cgatGCGGCCGCTAAAACAGAAGGGAACCTGGAGCAAGCCAAC	tcagTCTAGA*TTATTA*ATTTCCTTCTTCTGGGATGGTGAAAATGAG	To clone in IQ-A$_{1.4}$
Venus-IQ-A$_{1.2}$ (pcDNA3)	cgatGCGGCCGCTAAAACAGAAGGAAACCTGGAACAAGCCAAT	tcagTCTAGA*TTATTA*GTGGCCCTCCACGGTGCTGACTGTGCTGGG	To clone in IQ-A$_{1.2}$
Cerulean-ICDI$_{1.4}$ (pcDNA3)	cttaGCGGCCGCCTGCACGTGCCTGGAACCCACTC	gccgTCTAGA*CTATCA*GAGGGCGTGGACGCAGGCCATCTCG	To clone in ICDI$_{1.4}$
Cerulean-ICDI$_{1.2}$ (pcDNA3)	cgatGCGGCCGCTCTCACTGTGCCCAGCCAG	tcagTCTAGA*CTATCA*CAGGCTGCTGACGCCGGCCC	To clone in ICDI$_{1.2}$
Cerulean-ICDI$_{1.3}$ (pcDNA3)	cgatGCGGCCGCTCTGACCGTCCCCAGCAGCTTC	tcagTCTAGA*CTACTA*CAAGGTGGTGATGCAAATCATTTCATCTGCCAG	To clone in ICDI$_{1.3}$

Supplementary Table 3 | Primer list Underline: restriction sites; lower case: buffers of restriction sites; upper case: annealing to protein of interest; italic: special elements such as kozak (GCCACC) and stop codon (TAA, TGA, TAG), P2A sequence.

Supplementary References

1. Sang L, Bazzazi H, Ben Johny M, Yue DT. Resolving the Grip of the Distal Carboxy Tail on the Proximal Calmodulatory Region of CaV Channels (abstr.). *Biophysical Journal* **102**, 126a-126a (2012).

2. Depry C, Allen MD, Zhang J. Visualization of PKA activity in plasma membrane microdomains. *Molecular bioSystems* **7**, 52-58 (2011).
